# Establishment of Prognosis Model in Acute Myeloid Leukemia Based on Hypoxia Microenvironment, and Exploration of Hypoxia-Related Mechanisms

**DOI:** 10.3389/fgene.2021.727392

**Published:** 2021-10-26

**Authors:** Jinman Zhong, Hang Wu, Xiaoyin Bu, Weiru Li, Shengchun Cai, Meixue Du, Ya Gao, Baohong Ping

**Affiliations:** ^1^ Department of Hematology, Nanfang Hospital, Southern Medical University, Guangzhou, China; ^2^ Department of Huiqiao, Nanfang Hospital, Southern Medical University, Guangzhou, China

**Keywords:** acute myeloid leukemia, prognostic model, hypoxia, metabolism, immunity, bone marrow microenvironment

## Abstract

Acute myeloid leukemia (AML) is a highly heterogeneous hematologic neoplasm with poor survival outcomes. However, the routine clinical features are not sufficient to accurately predict the prognosis of AML. The expression of hypoxia-related genes was associated with survival outcomes of a variety of hematologic and lymphoid neoplasms. We established an 18-gene signature-based hypoxia-related prognosis model (HPM) and a complex model that consisted of the HPM and clinical risk factors using machine learning methods. Both two models were able to effectively predict the survival of AML patients, which might contribute to improving risk classification. Differentially expressed genes analysis, Gene Ontology (GO) categories, and Kyoto Encyclopedia of Genes and Genomes (KEGG) pathway enrichment analysis were performed to reveal the underlying functions and pathways implicated in AML development. To explore hypoxia-related changes in the bone marrow immune microenvironment, we used CIBERSORT to calculate and compare the proportion of 22 immune cells between the two groups with high and low hypoxia-risk scores. Enrichment analysis and immune cell composition analysis indicated that the biological processes and molecular functions of drug metabolism, angiogenesis, and immune cell infiltration of bone marrow play a role in the occurrence and development of AML, which might help us to evaluate several hypoxia-related metabolic and immune targets for AML therapy.

## Introduction

Acute myeloid leukemia (AML) is a clonal malignant aggressive hematological tumor, resulting in the accumulation of acquired chromosomal, genetic, and epigenetic abnormalities in highly heterogeneous myeloid precursors. It is the most common acute leukemia and accounts for approximately 80% of cases in adults. In the United States, the age-adjusted incidence of AML is 4.3 per 100,000 population annually (Shallis et al., 2019), which has a high mortality rate and variable prognosis. In recent years, the incidence rate of AML is getting increasingly serious and poses an enormous threat to human health. In the research of AML, there remain several challenges, advances in treatment for AML have remained quite limited, and the current prognostic evaluation system cannot completely distinguish the prognosis of AML patients. One of the hotspots and critical points for medical research is to identify specific prognostic factors that may help predict the outcomes.

Hypoxia is a common condition in the solid-tumor microenvironment (Kim et al., 2007), playing an important role in various biological processes, such as metabolic alteration, angiogenesis, and metastasis (Cosse and Michiels, 2008; Godet et al., 2019). However, the role of the pathophysiological implications of hypoxia in AML remains controversial, and the mechanism is still not clear. In bone marrow (BM), the low oxygen partial pressure (pO_2_) is physiological (Harrison et al., 2002). And the hypoxic microenvironmental niches within leukemic BM compared with those of the normal BM were expanded, accompanied by leukemia stem cell (LSC) proliferation (Keith and Simon, 2007; Benito et al., 2011; Benito et al., 2013; Zhou et al., 2016). The hypoxic BM microenvironment has also been shown to contribute to acute leukemic progression, resistance to chemotherapy, and minimal residual disease (MRD) (Frolova et al., 2012; Matsunaga et al., 2012; Tabe and Konopleva, 2015). In response to hypoxia, cells change their hypoxia-related gene expression, which was proved to be correlated with prognosis for various solid tumors.

However, the European LeukemiaNet (ELN) 2017 risk classification (ELN 2017) (Dohner et al., 2017), an important AML risk stratification standard that has been widely used to estimate prognosis of AML, is based on cytogenetic and molecular features. Mutations in the FMS-like tyrosine kinase 3 gene (FLT3-ITD) are quite common in AML and have been associated with poorer overall survival (OS) (Kayser et al., 2009). Nucleophosmin (NPM1) gene mutations have been associated with improved outcomes in patients with AML (Becker et al., 2010). Mutations of the CCAAT/enhancer binding protein alpha (CEBPA) gene have been associated with a favorable outcome in patients with AML, but mainly in those patients with cytogenetically normal AML (Renneville et al., 2009; Rockova et al., 2011). Rare studies to date have developed a hypoxia-related prognosis model (HPM) of AML based on gene expression profiles. AML-suitable hypoxia gene signatures still need to be developed.

To evaluate the potential utility of hypoxia-related gene expression profiles in AML prognosis, The Cancer Genome Atlas (TCGA) and MsigDB databases were analyzed, and the clinical features of patients were considered to construct an 18-gene-based hypoxia risk classifier. The model could be useful for the prognostic evaluations and development of novel therapeutic modalities aimed at interfering with hypoxia-sensing pathways and modifying the hematopoietic microenvironment.

## Materials and Methods

### Acquiring and Pre-Processing of Sample Data and Primary Screening of Acute Myeloid Leukemia Hypoxia-Related Genes

In this study, three public accessible transcriptome datasets of BEATAML1.0 (Cohort 1, https://portal.gdc.cancer.gov/projects/BEATAML1.0-COHORT; Supplementary Data Sheet S1), TARGET-AML (Cohort 2, https://portal.gdc.cancer.gov/projects/TARGET-AML; Supplementary Data Sheet S2), and TCGA-LAML (Cohort 3, https://portal.gdc.cancer.gov/projects/TCGA-LAML; Supplementary Data Sheet S3) were used throughout the training and validation stages. The latest clinical follow-up information was also obtained from Vizome (http://www.vizome.org/; Supplementary Data Sheet S4) (Tyner et al., 2018) or TCGA database (https://cancergenome.nih.gov/; Supplementary Data Sheet S5, S6). A total of 315 hypoxia-related genes defined in the Molecular Signatures Database (http://www.gsea-msigdb.org/gsea/msigdb/; Supplementary Table S1) were used as the initial candidates for Cox and least absolute shrinkage and selection operator (LASSO) survival analysis. We applied strict quality control (QC) for these datasets on sample and gene levels, respectively. On the sample level, we removed patients diagnosed with myelodysplastic syndromes (MDSs), myeloproliferative neoplasms (MPNs), or other non-AML diseases; we also filtered out individuals with no survival information. On the gene level QC, we kept genes having expression information in all three transcriptome datasets; genes with low expression quantity in all samples [reads per kilobase of transcript per million mapped reads (RPKM) < 1] were removed from downstream analysis. The principal component analysis (PCA) was also performed to identify the outlier; we removed individuals who deviated from the study samples. The ComBat method was used to correct the potential batch effects of RNA sequencing (Supplementary Figure S1). At this point, a total of 419 samples in Cohort 1, 156 samples in Cohort 2, and 151 samples in Cohort 3 (315 hypoxia-related genes) were kept for survival analysis (Supplementary Data Sheet S7–S9).

### Identification of Hypoxia-Related Signatures and Establishment and Verification of a Hypoxia Risk Score Model

Univariate Cox proportional hazards regression was first used to preliminarily screen the AML prognostic genes (*p* < 0.05). Next, Cohort 1 was randomly divided into a training set of 293 cases and a test set of 126 cases (7:3 ratio). To narrow down the prognostic genes for prediction, a Cox proportional hazards regression model combined with the LASSO (Goeman, 2010) using the “glmnet” package was applied to select the most important hypoxia-related signatures, and the optimal values of the penalty parameter λ were determined by 10-fold cross-validations at which the minimal mean squared error (MSE) is achieved in the training set (Simon et al., 2011). Afterward, the multivariate Cox regression analysis was performed to estimate independent prognostic factors associated with patient survival. Finally, the stepwise method was employed to select the best subset of predictors in a risk score model. To this, a hypoxia-related prognostic risk (HRS) score model was built, with the regression coefficients (β) weighted by the multivariate Cox proportional hazards regression model in the training set. The HRS model formula was as follows:

$$Hypoxia\;risk\;score=\overset{n}{\underset{i=1}{\sum }}\left(\beta_{i}\;*\;x_{i}\right)$$

β_i_ are coefficients (β) weighted by the multivariate Cox proportional hazards regression model, and x_i_ is the RNA expression level.

We calculate the HRS of the study samples and use the median HRS of the training group as the cutoff point to label the low-risk or high-risk individual of the three cohorts. The trained model was then tested using the test and validation sets (other independent cohorts: Cohort 2 and Cohort 3). The “survminer” package was used for the Kaplan–Meier (KM) survival analysis of patients in the high-risk and low-risk groups, while the “timeROC” package was used to construct time-dependent receiver operating characteristic (ROC) curves and calculate the area under the ROC curve (AUC) at 1-, 3-, and 5-years OS.

### Construction and Evaluation of the Nomogram Model

We explored the relationship between HPM and other clinical parameters for AML patient outcomes. Univariable Cox analysis and multivariate Cox analysis were performed with all patients’ clinical covariates in the BEATAML1.0 cohort by the “rms” package. Samples with incomplete data about potential prognostic factors were excluded from the multivariable Cox analyses. Pearson’s correlation between HPM and different clinical characteristics was calculated by “stats” package and plotted by “corplot” package. A nomogram was formulated using the “rms” r package based on the results of the multivariate analyses; and calibration plots and time-dependent ROC plots were performed to assess the prognostic accuracy of the nomogram. The predicted outcomes and observed outcomes of the nomogram were plotted in the calibration curve to evaluate the degree of fitting of the nomogram, and the 45° line represented the best prediction.

### Biological Phenotypes Associated With the Hypoxia Risk Score Model

#### Hypoxia-Related Metabolic Alterations

We procured 3,695 human metabolic genes concerning 145 metabolic subsystems from the Recon 3D (http://vmh.life) (Brunk et al., 2018). Among them, 3,224 metabolic genes were matched to our Cohort 1 data. The empirical Bayes algorithm of the R package “limma” (Ritchie et al., 2015) was used to identify differentially expressed genes (DEGs) between the top 25% samples of the high- and low-risk groups (totally 210 samples). All gene expression values were log2 transformed to identify the metabolic genes with significant differential expression during hypoxia stress [logarithmically transformed fold change (log2(FC)) ≥ 1 or (log2(FC)) ≤ −1 and *p*-value < 0.05]. Besides, we performed Kyoto Encyclopedia of Genes and Genomes (KEGG) and Gene Ontology (GO) enrichment analyses of DEGs with clusterProfiler package (Yu et al., 2012). KEGG pathway enrichment analysis utilized the KEGG database (http://www.genome.jp/kegg) while GO enrichment was utilized (http://www.geneontology.org).

#### Hypoxia-Related Immune Alterations

Regarding the association between the hypoxia risk score and immune cells in the BM microenvironment, CIBERSORT algorithm (Newman et al., 2015) was used to estimate the relative immune cell fractions in the BM samples of Cohort 1, based on the standard LM22 leukocyte signature matrix that distinguishes 22 immune cell subtypes and 1,000 permutations (CIBERSORT R script v1.03 is available on http://cibersort.stanford.edu/). We performed the following analyses in CIBERSORT: B cells, CD4^+^ T cells, CD8^+^ T cells, Tregs, NK T cells, γδ-T cells, lymphocytes, and macrophages. Further, xCell (http://xcell.ucsf.edu/) was used to validate the result.

## Result

**FIGURE 1:**
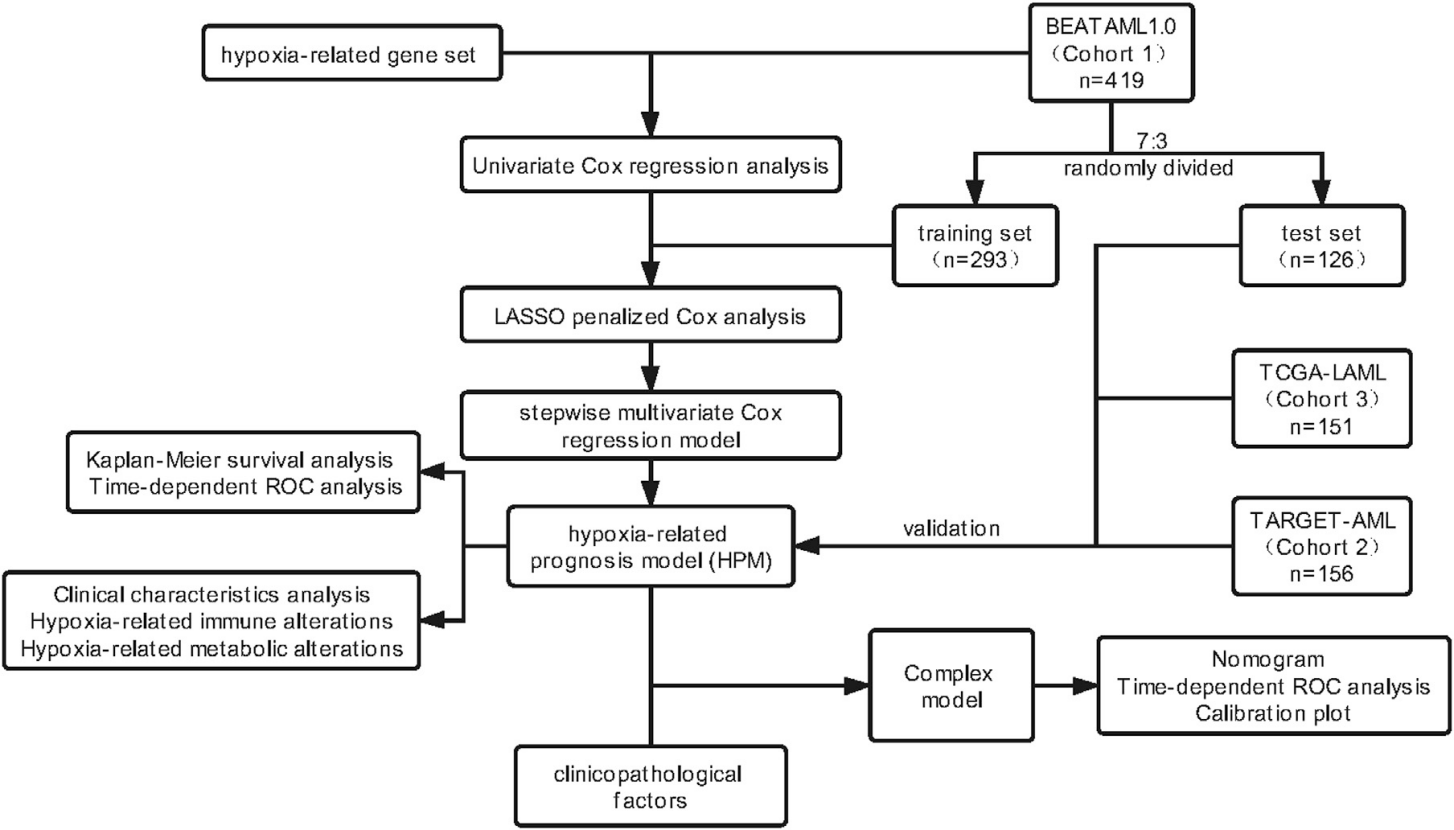
Flowchart of data collection, modeling, and further analysis.

A flowchart overviewing the procedures of this study is presented in Figure 1.

### Patient Clinical Characteristics

We analyzed clinical characteristics of patients with AML from Cohort 1, including age, gender, ELN 2017, the mutations of NPM1 and FLT3, and CEBPA Biallelic status (Table 1). Differences in general clinical information of two sets are not statistically significant. Among the 419 AML patients with complete clinical information in BEATAML1.0, the mean diagnosis age was 56.22 ± 18.23 years, while the proportion of males was 55.61% (233/419).

**TABLE 1:** Patients’ basic characteristics in BEATAML1.0 cohort.

		Total *n* = 419	Training set *n* = 293	Test set *n* = 126	*p*-value
Age (mean (SD)) (%)		56.22 (18.25)	55.98 (18.29)	56.77 (18.23)	0.685
	<65	256 (61.1)	185 (63.1)	71 (56.35)	0.231
	≥65	163 (38.9)	108 (36.9)	55 (43.7)	
Gender (%)					0.582
	Female	186 (44.4)	127 (43.3)	59 (46.8)	
	Male	233 (55.6)	166 (56.7)	67 (53.2)	
ELN 2017 (%)					0.846
	Favorable	108 (25.8)	77 (26.3)	31 (24.6)	
	Intermediate	140 (33.4)	93 (31.7)	47 (37.3)	
	Adverse	149 (35.6)	108 (36.9)	41 (32.5)	
	Favorable or intermediate	14 (3.3)	9 (3.1)	5 (4.0)	
	Intermediate or adverse	7 (1.7)	5 (1.7)	2 (1.6)	
	Not available	1 (0.2)	1 (0.3)	0 (0.0)	
NPM1 (%)					0.439
	Negative	309 (73.7)	213 (72.7)	96 (76.2)	
	Positive	107 (25.5)	77 (26.3)	30 (23.8)	
	Not available	3 (0.7)	3 (1.0)	0 (0.0)	
FLT3-ITD (%)					0.233
	Negative	321 (76.6)	218 (74.4)	103 (81.7)	
	Positive	97 (23.2)	74 (25.3)	23 (18.3)	
	Not available	1 (0.2)	1 (0.3)	0 (0.0)	
CEBPA Biallelic (%)					1
	No	412 (98.3)	124 (98.4)	288 (98.3)	
	Yes	7 (1.7)	2 (1.6)	5 (1.7)	

### Establishment of Hypoxia Risk Score Model

**FIGURE 2:**
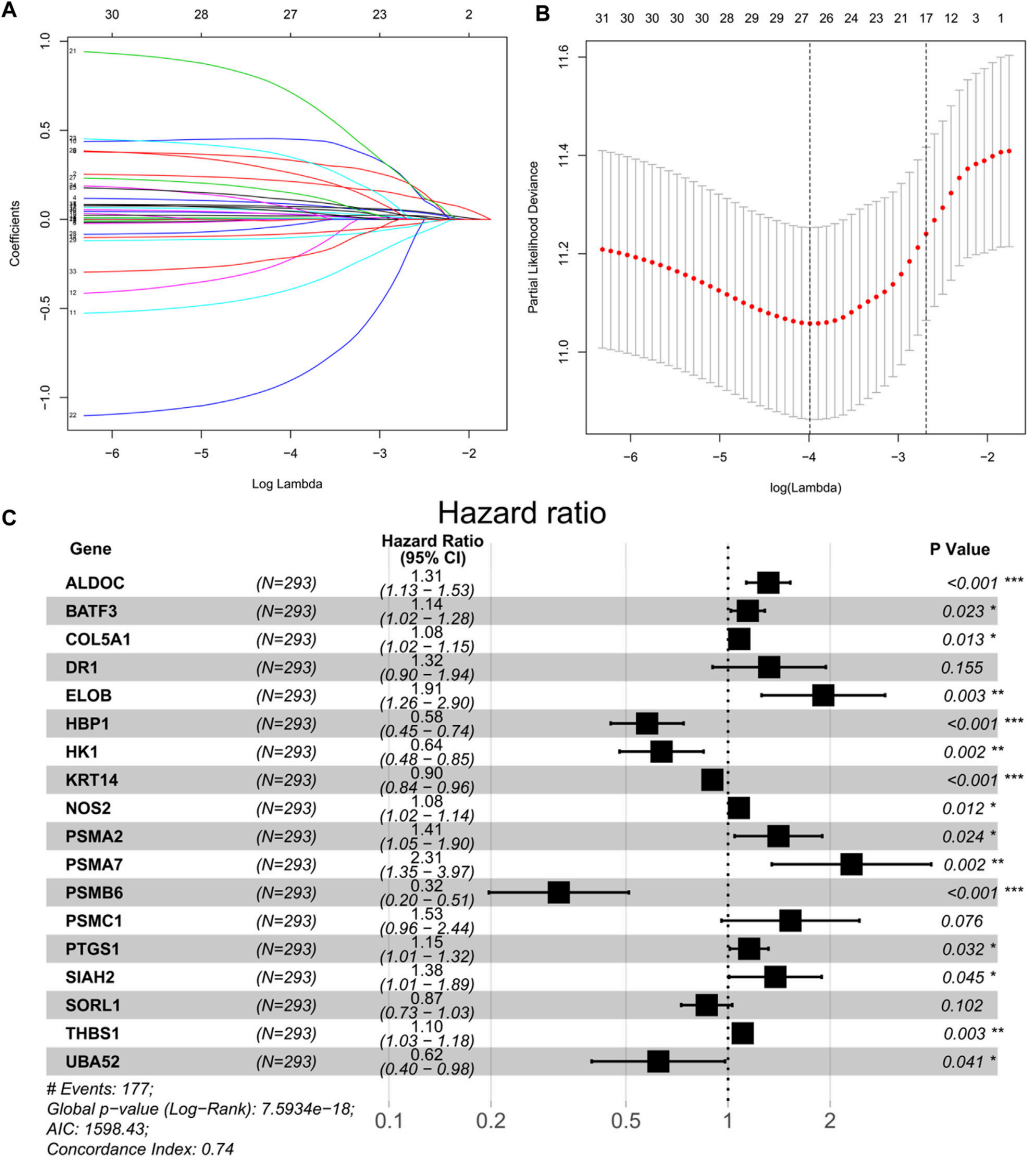
**(A)** Tenfold cross-validation for tuning parameter selection in the LASSO model. **(B)** Least absolute shrinkage and selection operator (LASSO) coefficient profiles of the 33 prognostic genes. **(C)** Forest plot of 18 hypoxia-related genes significantly associated with overall survival according to multivariate Cox regression analysis.

A univariate Cox regression was performed to identify prognostic hypoxia-related genes associated with OS in BEATAML1.0 dataset. A total of 33 genes (*ALDH1A1*, *ALDOC*, *BACE2*, *BATF3*, *CA9*, *CALD1*, *COL5A1*, *DR1*, *EGLN3*, *ELOB*, *HBP1*, *HK1*, *ID2*, *KRT14*, *LRP8*, *NOS1*, *NOS2*, *PDK3*, *PLOD2*, *PSMA2*, *PSMA7*, *PSMB6*, *PSMC1*, *PSMC4*, *PTGS1*, *RPS27A*, *SIAH2*, *SLC16A1*, *SORL1*, *TGM2*, *THBS1*, *TPD52*, and *UBA52*) were identified to have a significant prognostic value in patients with AML (*p* < 0.05). Then, LASSO-penalized Cox analysis with 10-fold cross-validation (Figures 2A,B) was performed for further screening, and 23 genes were left. Finally, a total of 18 hub genes (*ALDOC*, *BATF3*, *COL5A1*, *DR1*, *ELOB*, *HBP1*, *HK1*, *KRT14*, *NOS2*, *PSMA2*, *PSMA7*, *PSMB6*, *PSMC1*, *PTGS1*, *SIAH2*, *SORL1*, *THBS1*, and *UBA52*) were identified from the stepwise multivariate Cox regression (Figure 2C), and the formula to calculate the hypoxia risk score was as follows:

$$\begin{array}{l} \text{Hypoxia}\;\text{risk}\;\text{score}=\text{0}\text{.}2737\times \text{ALDOC}+\text{0}\text{.}1343\times \text{BATF}3\times \text{0}\text{.}0762\times \text{COL}5\text{A}1+\text{0}\text{.}2795\times \text{DR}1+\text{0}\text{.}6469\times \text{ELOB}-\text{0}\text{.}5492\times \text{HBP}1-\text{0}\text{.}4513\times \text{HK}1-\text{0}\text{.}1064\times \text{KRT}14\\ +\text{0}\text{.}0740\times \text{NOS}2+\text{0}\text{.}3421\times \text{PSMA}2+\text{0}\text{.}8385\times \text{PSMA}7-1\text{.}1489\times \text{PSMB}6+\text{0}\text{.}4238\times \text{PSMC}1+\text{0}\text{.}1434\times \text{PTGS}1+\text{0}\text{.}3213\times \text{SIAH}2-\text{0}\text{.}1447\times \text{SORL}1+\text{0}\text{.}0994\times \\ \text{THBS}1-\text{0}\text{.}4732\times \text{UBA}52 \end{array}$$

### Prognostic Value of the Hypoxia Risk Score

**FIGURE 3:**
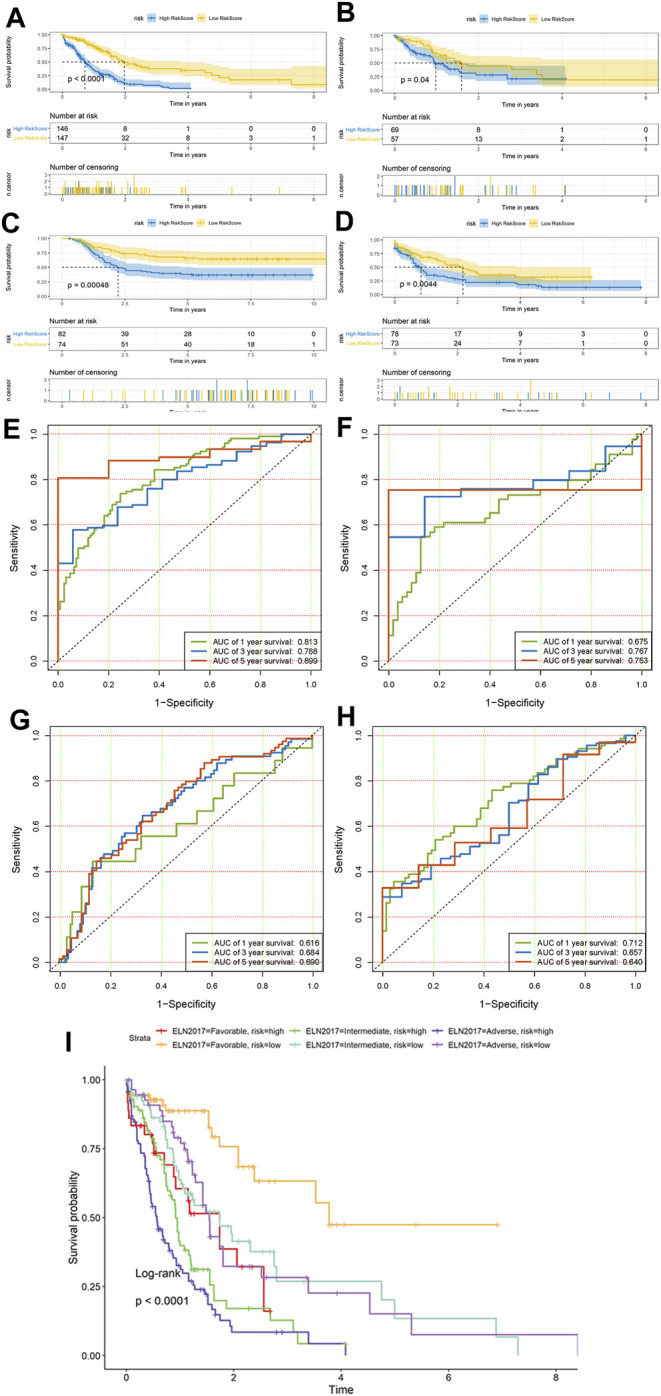
Prognostic value of the hypoxia risk score Kaplan–Meier survival curves of overall survival (OS) between the low- and high-risk group patients in all three cohorts. **(A)** Training dataset of Cohort 1 and **(B)** test dataset of Cohort 1, **(C)** Cohort 2, and **(D)** Cohort 3. ROC curves of the hypoxia risk score model based on the 18 characteristic genes. **(E)** Training dataset of Cohort 1 and **(F)** test dataset of Cohort 1, **(G)** Cohort 2, and **(H)** Cohort 3. ROC, receiver operating characteristic; AUC, area under the curve. **(I)** Comparison of survival between six different ELN2017 and hypoxia-related prognosis model (HPM) risk subgroups of patients.

The KM survival curves revealed that patients in the high-risk group exhibited a significantly lower OS rate than the low-risk group in all cohorts (training set, test set, Cohort 2, and Cohort 3; *p* < 0.0001, *p* = 0.04, *p* < 0.001, and *p* < 0.01, respectively; Figure 3). In the training dataset of BEATAML1.0 Cohort, the median OS of low-risk patients was 1.970 years (95% CI: 1.734–3.775), whereas the median OS of high-risk patients was 0.718 years (95% CI: 0.575–0.912), and the HR is 3.261 (95% CI: 2.398–4.435). In comparison, in the test dataset of BEATAML1.0 Cohort (validation Cohort 1), the median OS of patients with low-risk scores was 1.592 years (95% CI: 1.230–NA), and the median OS of patients with high-risk scores was 0.978 years (95% CI: 0.860–1.556), while HR is 1.626 (95% CI: 1.007–2.626). In TARGET-AML cohort (validation Cohort 2), the median OS of patients with low-risk scores was NR (not reached), and the median OS of patients with high-risk scores was 2.227 years (95% CI: 1.689–5.195), and the HR is 2.283 (95% CI: 1.455–3.582). In TCGA-LAML cohort (validation Cohort 3), the median OS of patients with low-risk scores was 2.170 years (95% CI: 1.581–3.838), the median OS of patients with high-risk scores was 0.833 years (95% CI: 0.586–1.003), and the HR is 1.773 (95% CI: 1.190–2.642). Furthermore, the prognostic accuracy of the hypoxia risk score was assessed with time-dependent ROC analysis for OS at 1, 3, and 5 years in all datasets, and the results were as follows. In the training set, the AUC was 0.813 at 1 year, 0.788 at 3 years, and 0.899 at 5 years (Figure 3E); 0.675 at 1 year, 0.767 at 3 years, and 0.753 at 5 years (Figure 3F) in the test set; 0.616 at 1 year, 0.684 at 3 years, and 0.690 at 5 years (Figure 3G) in Cohort 2; and 0.712 at 1 year, 0.657 at 3 years, and 0.640 at 5 years (Figure 3H) in Cohort 3, which revealed that the hypoxia risk score was a valuable predictor. We next studied whether the HRS could improve prognostic assessment in AML patients on the 2017 ELN genetic risk stratification (Dohner et al., 2017) basis. In each ELN2017 stratification, the HPM high-risk group had a poorer prognosis than the HPM low-risk group (Figure 3I). Furthermore, we found that 37 out of 108 ELN2017 favorable patients (51.7%) were high-risk for the HPM and had significantly worse survival. In this patient subgroup (ELN favorable–HPM high), representing 9.3% (37/397, only contains patients who had clear ELN2017 stratification) of the BEATAML1.0 cohort, their survival was similar to that of ELN intermediate/adverse-risk patients with HPM low-risk. To further evaluate the performance of the HPM, we compared our HPM with other gene expression based AML prognostic models, which were published within the last 5 years, including PMID29138577 (Huang et al., 2017), PMID32268820 (Zhang and Xiao, 2020), PMID29956722 (Zhao et al., 2018), and PMID34282207 (Jiang et al., 2021). Risk score was calculated based on formulas from the corresponding literatures. We used the *p*-value of the KM survival analysis to reflect the discrimination and the AUC value to evaluate the accuracy. The survival analysis showed that the HPM was significantly correlated with the survival of the patients in all three datasets. In contrast, other models could only perform well in at most two of the three datasets (Supplementary Figure S2). The AUC values of the HPM were more stable, which means a wider range of suitable population (Supplementary Figure S3; Supplementary Table S2).

### Hypoxia-Related Characteristics of High- and Low-Risk Patients, Based on the Prognostic Risk Score Model

#### Association Between the Hypoxia Risk Score and the Clinical Characteristics

**FIGURE 4:**
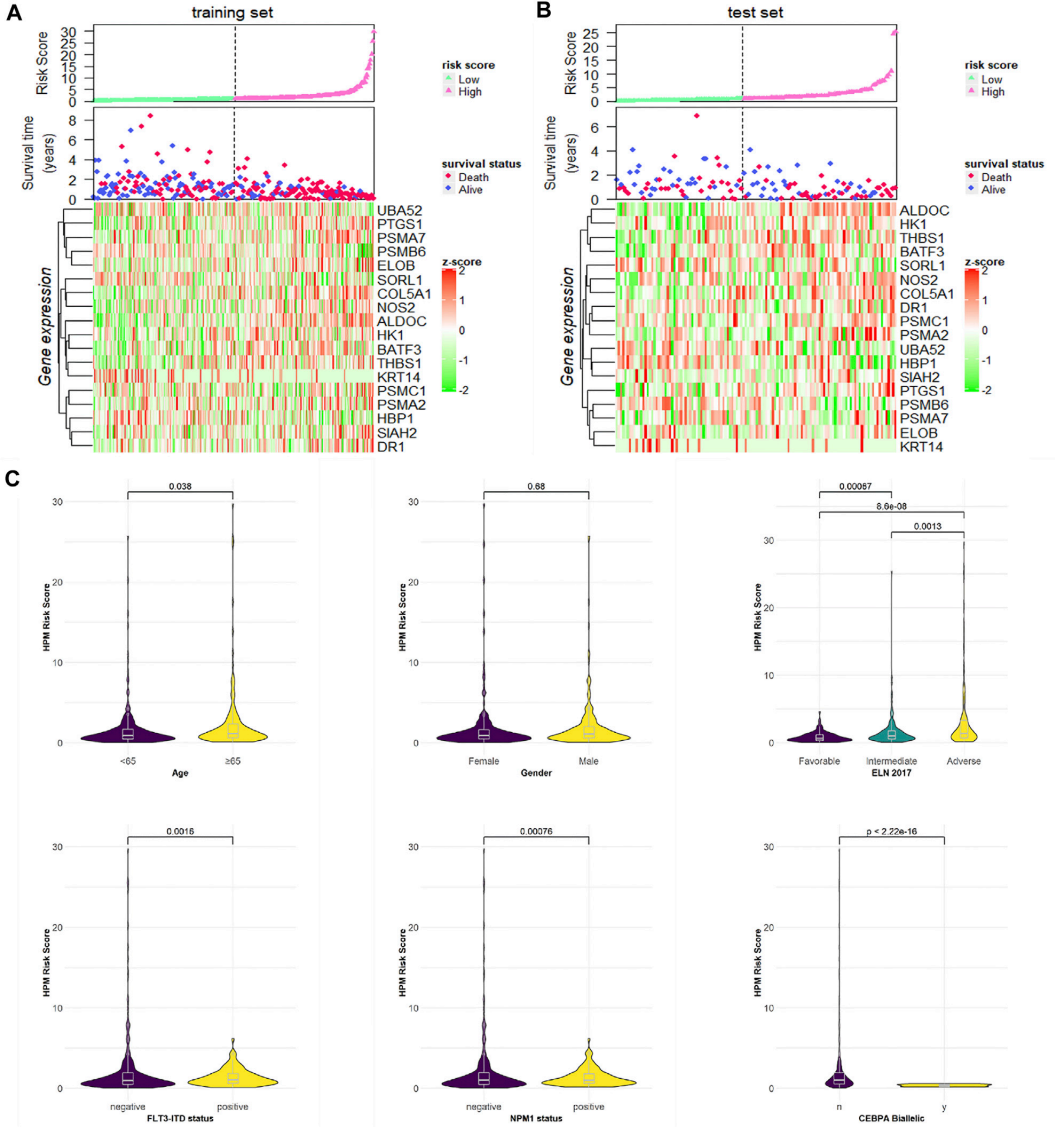
Risk score distributions, genes expression, survival time, and status profiles in the **(A)** training and **(B)** test set. **(C)** Hypoxia risk score group by stratification factors.

We ranked the risk scores of patients in the training and test sets, and the distributions associated with gene expression, survival time, and status are shown in Figures 4A,B. Hypoxia risk score increased with higher age, worse 2017 ELN genetic risk stratification, NPM1 wild type, and FLT3-ITD wild type and have no CEBPA double mutation (Figure 4C).

#### Hypoxia-Related Prognosis Model-Related Metabolic Alterations

**FIGURE 5:**
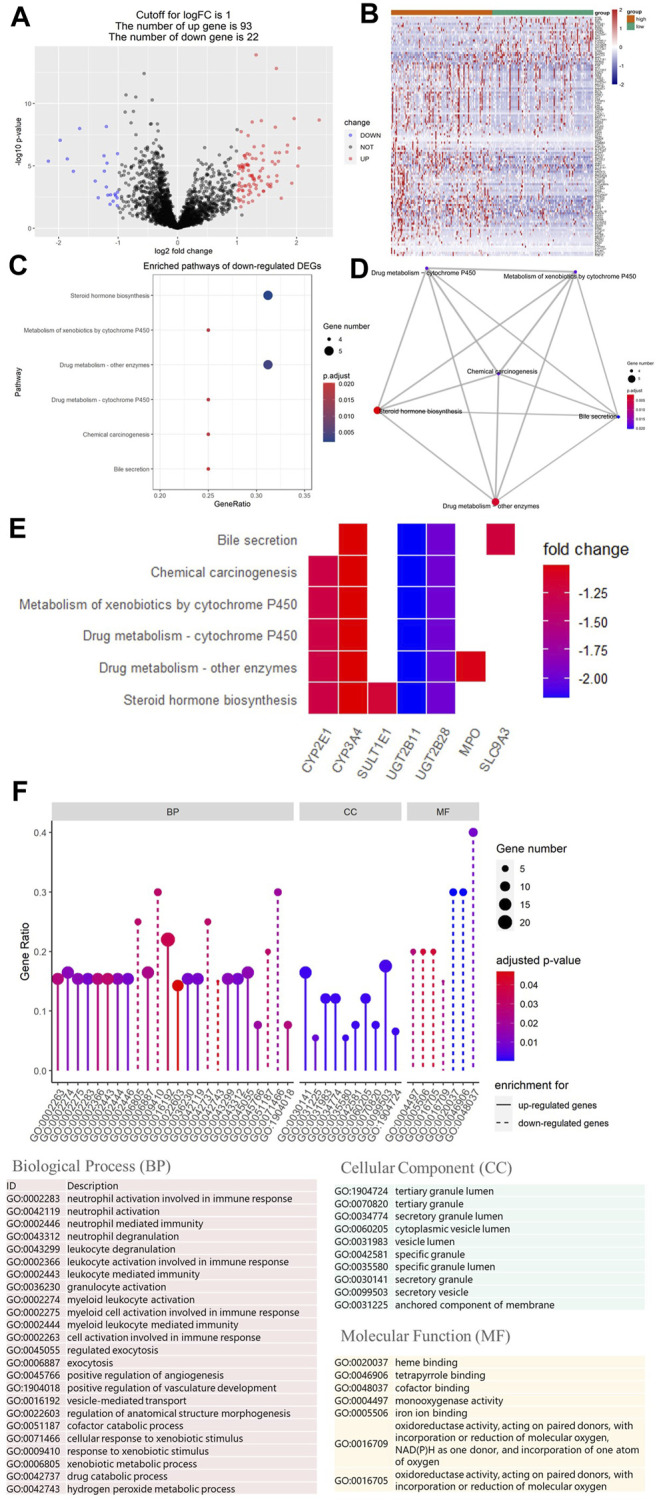
Differential gene expression analysis of metabolic genes. And Kyoto Encyclopedia of Genes and Genomes (KEGG) and Gene Ontology (GO) analysis of differentially expressed genes (DEGs). **(A)** Volcano plot showing DEGs between the top 25% samples of the high- and low-risk groups. Each dot represents one gene. Red dot represents upregulated gene (log2 (fold change) > 1 and *p*-value < 0.05). Blue dot represents downregulated gene (log2 (fold change) < −1 and *p*-value < 0.05). And black dot represents non-differentially expressed gene. **(B)** Heatmap of DEGs associated with hypoxia-related prognosis model (HPM) risk group. **(C)** Bubble graph of the enrichment KEGG pathways for the downregulated genes (there are no significantly enriched KEGG pathways for upregulated genes). **(D)** Enrichment map of the enrichment KEGG pathways for the downregulated genes showing the association between different pathways. **(E)** Heatmap of the enrichment KEGG pathways for the downregulated genes, showing the association between pathways and genes. **(F)** Lollipop plot of GO term enrichment for Biological Process, Cellular Component, and Molecular Function. In the figure, the size of the dot indicates the number of DEGs that were enriched in the pathway, the color of the dot corresponds to the different *p*-values, and the solid line is used for GO terms enriched for upregulated genes, while dashed line is used for GO terms enriched for downregulated genes.

To study the relationship between HRS and metabolic flux, we performed differential gene expression analysis using “limma” package, 115 DEGs were shortlisted from the raw dataset, and 93 genes were upregulated in the high-risk group while 22 genes were downregulated. Results were visualized into volcano plot (Figure 5A) and heatmap (Figure 5B). To evaluate the molecular mechanisms of DEGs, KEGG metabolic pathways enrichment analyses, and GO functional annotation were conducted (Table 2; Figure 5). Unsurprisingly, pathways associated with oxidoreduction activity were enriched. Unexpectedly, we found 12 immune-related pathways such as “leukocyte mediated immunity” and “cell activation involved in immune response,” which were significantly overrepresented. Besides, two pathways of angiogenesis were significantly enriched in the upregulated genes for the high-risk group. And the enrichment of “heme binding” and “tetrapyrrole binding” might be associated with oxygen-carrying capacity. Moreover, drug catabolic process pathways were markedly enriched, such as “xenobiotic metabolic process,” “drug catabolic process,” “Drug metabolism—other enzymes,” “Drug metabolism—cytochrome P450,” and “Metabolism of xenobiotics by cytochrome P450.” Moreover, the enrichment of the pathway “Chemical carcinogenesis” suggests different disease susceptibility between the high- and low-risk groups.

**TABLE 2:** KEGG pathway enrichment analyses and GO functional annotation result.

Terms	ID	Description
KEGG	hsa00140	Steroid hormone biosynthesis
	hsa00983	Drug metabolism—other enzymes
	hsa00982	Drug metabolism—cytochrome P450
	hsa00980	Metabolism of xenobiotics by cytochrome P450
	hsa05204	Chemical carcinogenesis
	hsa04976	Bile secretion
GO: BP	GO:0002283	Neutrophil activation involved in immune response
	GO:0042119	Neutrophil activation
	GO:0002446	Neutrophil mediated immunity
	GO:0043312	Neutrophil degranulation
	GO:0043299	Leukocyte degranulation
	GO:0002366	Leukocyte activation involved in immune response
	GO:0002443	Leukocyte mediated immunity
	GO:0036230	Granulocyte activation
	GO:0002274	Myeloid leukocyte activation
	GO:0002275	Myeloid cell activation involved in immune response
	GO:0002444	Myeloid leukocyte mediated immunity
	GO:0002263	Cell activation involved in immune response
	GO:0045055	Regulated exocytosis
	GO:0006887	Exocytosis
	GO:0045766	Positive regulation of angiogenesis
	GO:1904018	Positive regulation of vasculature development
	GO:0016192	Vesicle-mediated transport
	GO:0022603	Regulation of anatomical structure morphogenesis
	GO:0051187	Cofactor catabolic process
	GO:0071466	Cellular response to xenobiotic stimulus
	GO:0009410	Response to xenobiotic stimulus
	GO:0006805	Xenobiotic metabolic process
	GO:0042737	Drug catabolic process
	GO:0042743	Hydrogen peroxide metabolic process
GO: CC	GO:1904724	Tertiary granule lumen
	GO:0070820	Tertiary granule
	GO:0034774	Secretory granule lumen
	GO:0060205	Cytoplasmic vesicle lumen
	GO:0031983	Vesicle lumen
	GO:0042581	Specific granule
	GO:0035580	Specific granule lumen
	GO:0030141	Secretory granule
	GO:0099503	Secretory vesicle
	GO:0031225	Anchored component of membrane
GO: MF	GO:0020037	Heme binding
	GO:0046906	Tetrapyrrole binding
	GO:0048037	Cofactor binding
	GO:0004497	Monooxygenase activity
	GO:0005506	Iron ion binding
	GO:0016709	Oxidoreductase activity, acting on paired donors, with incorporation or reduction of molecular oxygen, NAD(P)H as one donor, and incorporation of one atom of oxygen
	GO:0016705	Oxidoreductase activity, acting on paired donors, with incorporation or reduction of molecular oxygen

Note: BP, biological process; CC, cellular component; GO, gene ontology; KEGG, kyoto encyclopedia of genes and genomes; MF, molecular function.

#### Hypoxia-Related Prognosis Model-Related Immune Alterations

**FIGURE 6:**
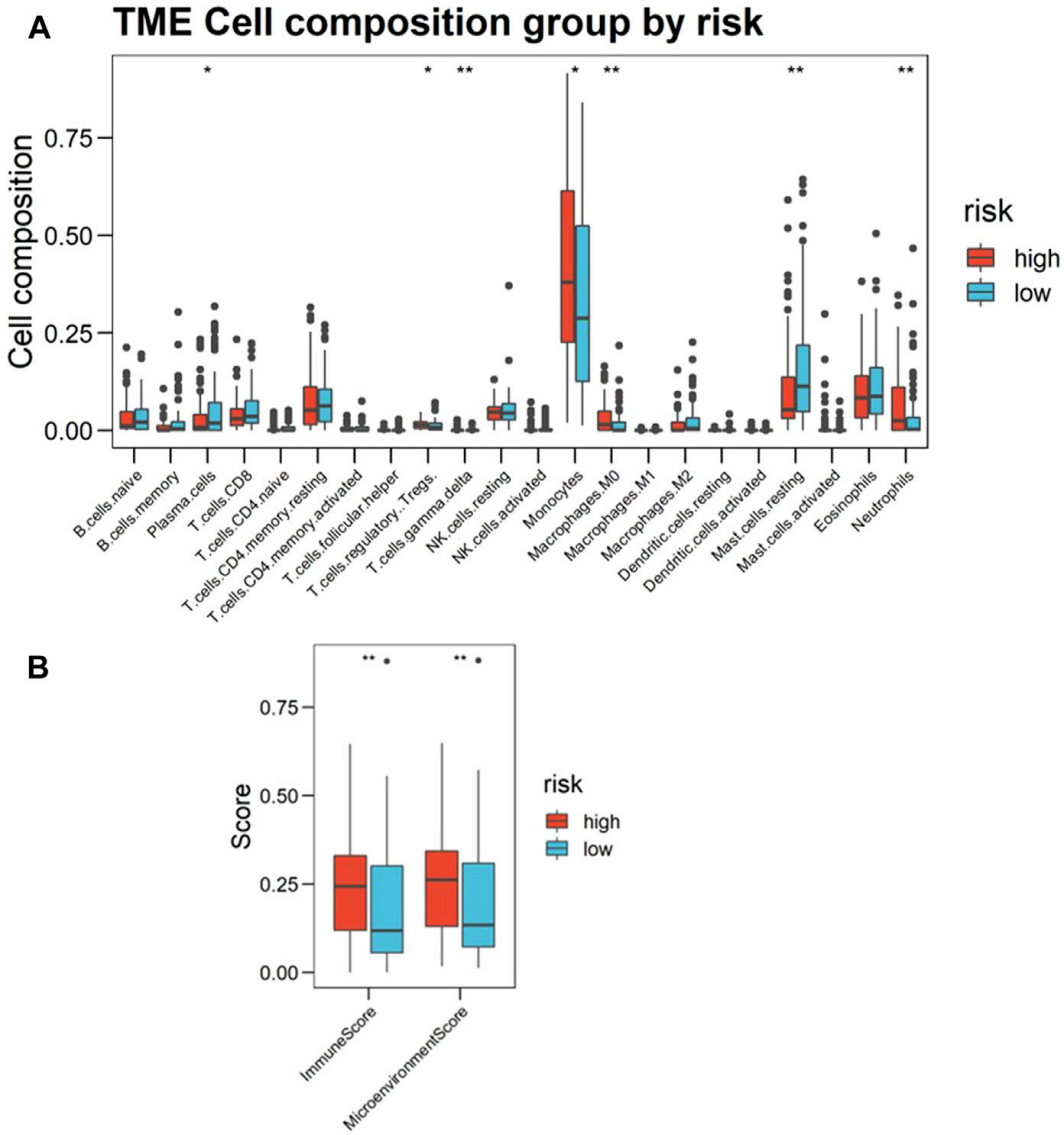
Landscape of immune infiltration in the bone marrow (BM) of acute myeloid leukemia (AML) patients, as estimated from gene-expression data (Cohort 1, BM samples *n* = 216) using CIBERSORT. **(A)** Boxplots visualizing significantly different immune cell infiltrations between high- and low-risk patients. The *p*-values calculated from Wilcoxon test are shown: *p-values < 0.05; **p-values < 0.01; ***p-values < 0.001. **(B)** Immune Score and Microenvironment Score between high- and low-risk patients scoring by xCell based on estimated immune cell proportion. Higher hypoxia risk is associated with higher Immune Score and Microenvironment Score.

**FIGURE 7:**
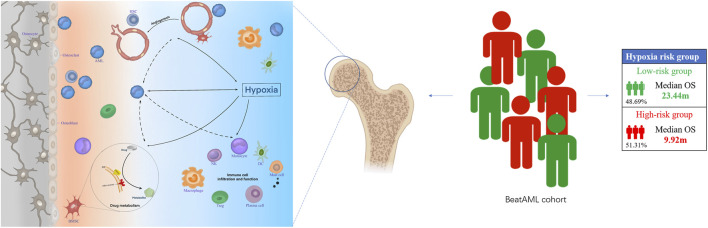
The hypoxia-related mechanisms that are involved in a poor prognosis of acute myeloid leukemia (AML) patients. Hypoxia may affect the prognosis of patients with AML by affecting angiogenesis, drug metabolism, and bone marrow immune microenvironment.

Based on the foregoing GO enrichment analysis results, we conjecture that hypoxia is related to immunity. Using CIBERSORT, we found the proportion of resting mast cells and plasma cells in the high-risk group significantly to be lower than that of the low-risk group, whereas the proportion of neutrophils, monocytes, M0 macrophages, γδ-T cells, and regulatory T cells (Tregs) increased in high-risk patients (Figure 6A; Supplementary Data Sheet S10). Cell types were also predicted and visualized using xCell, giving similar results. Further, xCell also shows that the Immune Score and Microenvironment Score of the high-risk group were significantly higher than of the low-risk group (Figure 6B). An overview of the predictive result of mechanism exploration is presented in Figure 7.

### A Complex Model for Prognostic Evaluation of Acute Myeloid Leukemia

**FIGURE 8:**
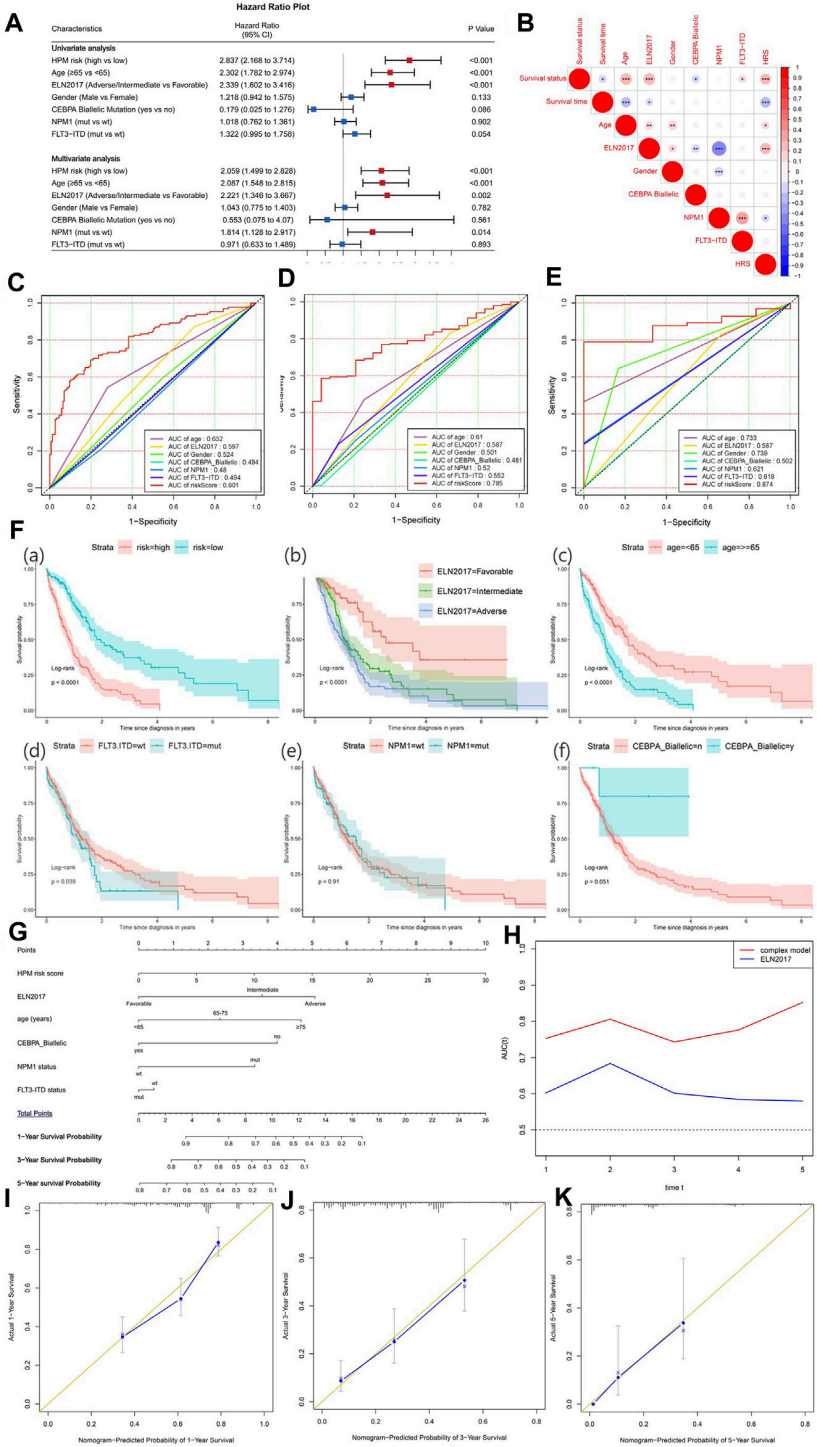
The establishment of a nomogram to predict the overall survival of acute myeloid leukemia (AML) patients. **(A)** Forest plot of univariate and multivariate Cox regression analysis of clinical characteristics in AML patients. The red dot with bar indicates statistical significance (*p* < 0.05). **(B)** Pearson’s correlation between hypoxia-related prognosis model (HPM) and different clinical characteristics (*p-values < 0.05; **p-values < 0.01; ***p-values < 0.001). Receiver operating characteristic (ROC) plot of HRM and important clinical characteristics at 1 **(C)**, 3 **(D)**, and 5 years **(E)**. **(F)** Survival analysis for HPM risk and other prognostic factors in AML. **(G)** Nomogram of the complex model for overall survival at 1, 3, and 5 years in AML patients. **(H)** Time-dependent ROC curves for complex model and ELN 2017. Calibration plot at 1 **(I)**, 3 **(J)**, and 5 years **(K)** for validation of prognostic nomogram.

To explore the independent prognostic factors for AML, univariate and multivariate Cox analyses were sequentially performed in the BEATAML1.0 dataset (Figure 8A), including the hypoxia risk score and other available clinical characteristics, such as age, gender, ELN 2017, the mutations of NPM1 and FLT3, and CEBPA Biallelic status. The HRS, ELN 2017, and age remained statistically significant (*p* < 0.05) in both the univariate and multivariate Cox analyses, indicating that HRS, ELN 2017, and age were independent prognostic factors. NPM1 status was an independent factor of prognosis after adjustment for other clinical factors. The correlation between clinical factors was analyzed and visualized in Figure 8B. HRS was positively correlated with survival status, age, and ELN2017 while negatively correlated with survival time and NPM1 status. Furthermore, Figures 8C–E indicate that the HRS had a higher AUC than other clinical factors in 1-, 3-, and 5-years survival prediction. Overall, these results demonstrated that the HPM can predict the AML prognosis independently and effectively. To reveal the prognostic value, maximize practicability, and facilitate clinicians’ usage of our model, we constructed a nomogram that was composed of both the hypoxia risk score and available clinical risk factors based on BEATAML1.0 cohort (Figure 8F). A combination of HRS and clinical risk factors was found to improve its prognostic value with a markedly better AUC than the 2017 ELN genetic risk stratification (Figure 8G). It is shown that the complex model can predict more accurately in the long term with the increasing tendency of AUC. To validate the predicted and actual probabilities at 1, 3, and 5 years, calibration plots were constructed (Figures 8H–J), and the nomogram performs well. These findings demonstrated that the nomogram is an optimal model for predicting the survival probability of AML patients than individual prognostic factors.

## Discussion

AML is a highly aggressive and heterogeneous hematologic malignancy (Döhner et al., 2015). Hypoxia is a significant outcome factor of leukemia patients, which could be reflected by the changed expression of related genes (Benito et al., 2016). Here, in our analysis, we identified an 18-hypoxia related gene-based prognosis model that could independently predict the survival probability of AML patients. To facilitate clinical application, we combined our HPM, 2017 ELN genetic risk stratification, and clinical risk factors to construct a complex model and nomogram, which outperformed 2017 ELN genetic risk stratification in the prediction of survival rate. Thus, the comprehensive nomogram could be utilized by clinicians in the near future. Further, we surmise that hypoxia may affect the prognosis of AML patients by affecting the angiogenesis, drug metabolism, and BM immune microenvironment.

The risk model created in this study consisted of 18 hypoxia-related genes, many of which were reported in cancer. ALDOC encodes a member of the class I fructose-biphosphate aldolase gene family and is involved in HIF-1 signaling pathway. ALDOC was identified as activators of Wnt signaling, a signaling pathway involved in cancer genesis and progression when it was over-activated (Caspi et al., 2014). Meanwhile, ALDOC was overexpressed in gallbladder carcinoma (Fan et al., 2020), melanoma (Izraely et al., 2020), and lung cancer (Yuan et al., 2021), associated with their growth or pathogenesis. BATF3 is an AP-1 family transcription factor that controls the differentiation of CD8(+) thymic conventional dendritic cells in the immune system. According to the KEGG database, it was involved in PD-L1 expression and PD-1 checkpoint pathway in cancer. Immunotherapy with immunomodulatory monoclonal antibodies targeting PD-1 or CD137 requires Batf3-dependent dendritic cells (Sanchez-Paulete et al., 2016). COL5A1 is a member of the fibrous subfamily of collagen. Overexpression of COL5A1 may promote metastasis of lung adenocarcinoma (Liu et al., 2018) and the progression of muscle-invasive bladder cancer (Ewald et al., 2013) and may increase the risk of hematogenous and lymphatic metastasis in serous ovarian cancer (Yue et al., 2019). COL5A1 is also overexpressed in gastric cancer, which may regulate the proliferation of gastric cancer cells by affecting the tumor microenvironment and is associated with poor prognosis (Wei et al., 2020). HBP1 plays a role in the regulation of the cell cycle and is a tumor suppressor (Bollaert et al., 2019). Downregulating HBP1 promotes the migration and invasion of oral squamous cell carcinoma (Li K. et al., 2020) and breast cancer (Li et al., 2011). HK1 encodes hexokinase 1, which is the first rate-limiting enzyme in glycolysis, is related to the progression of ovarian cancer (Li Y. et al., 2020) and colorectal cancer (Li S. et al., 2020). PSMA7 interacts with proteins such as HIF-1α, EMAP II, c-Abl, and Arg tyrosine kinases, which participated in tumorigenesis. Studies reported that PSMA7 expression was elevated in testicular, liver, breast, prostate, cervical, gastric, and colorectal cancers (Xia et al., 2019), while UBA52 is overexpressed in the colon (Barnard et al., 1995), and renal cancers (Kanayama et al., 1991). THBS1 was also implicated in the development of several cancers, including breast, gastric, melanoma, and cervical cancers and glioblastoma (Qi et al., 2021).

To probe into the mechanism of hypoxia in leukemia, differential gene expression analysis and enrichment analysis were implemented. The enriched biological process like “leukocyte mediated immunity” and “cell activation involved in immune response” suggested that hypoxia might affect cell-mediated immunity against cancer cells. Angiogenesis, which was particularly important for tumor survival in the hypoxic condition (Hanahan and Weinberg, 2011), was significantly enriched in the upregulated genes for the high-risk group. Although there are few studies about angiogenesis in hematological malignancy, BM angiogenesis in AML patients has been observed and may play a role in the pathogenesis (Hussong et al., 2000; Padro et al., 2000; Testa et al., 2020). BM microvessel density of AML patients is higher than that of healthy individuals at the time of diagnosis and decreases after remission (Padro et al., 2000; Song et al., 2015). The vascular niches could support the survival of leukemic cells and protect AML by regulating AML cell cycle through paracrine secretion and adhesive contact with endothelial cells, helping to resist chemotherapy (Cogle et al., 2016). Moreover, higher microvessel density at the time of diagnosis was associated with poor prognosis (Kuzu et al., 2004; Rabitsch et al., 2004). In addition, drug metabolism is significantly affected by changes in pharmacokinetics, expression, and function of drug metabolic enzymes and transporters under hypoxia (Donovan et al., 2010). Hypoxia affects the transcription and function of cytochrome P450 (CYP450) through HIF-1α (Min et al., 2019). CYP enzymes and other drug-metabolizing enzymes are expressed in BM stroma (Alonso et al., 2015). CYP450 is involved in the stroma-mediated resistance of AML cells to chemotherapy (Alonso et al., 2015). Drug catabolic process pathways, such as “xenobiotic metabolic process,” “drug catabolic process,” “Drug metabolism—other enzymes,” “Drug metabolism—cytochrome P450,” and “Metabolism of xenobiotics by cytochrome P450,” were markedly enriched, which might correlate with chemotherapeutic drugs concentration and sensitivity change in a hypoxic microenvironment of AML. Hypoxia might promote angiogenesis, disturb cell-mediated immunity balance, and affect pharmacokinetics to result in a bad prognosis.

To explore hypoxia-related changes in the BM immune microenvironment, immune cell composition analysis was performed. We found that the proportion of resting mast cells in the high-risk group was significantly lower than in the low-risk group, while activated mast cells in the high-risk group were higher than in the low-risk group, even though the difference did not reach statistical significance (mean proportion 0.12 vs. 0.02, *p* = 0.22). The present study shows that mast cells can promote cancer growth by stimulation of neoangiogenesis and modulation of the immune response (Dyduch et al., 2012). This might indicate that hypoxia affects angiogenesis and immune response by affecting the proportion of mast cells. The proportion of monocytes and M0 macrophages in the high-risk group were significantly higher than the low-risk group, which contribute to the poor prognosis of high-risk group patients. Tumor-infiltrating monocytes and macrophages have well-recognized facilitative roles in the initiation, migration, and invasion of solid tumors (Condeelis and Pollard, 2006; Qian and Pollard, 2010; Richards et al., 2013). Although not widely researched, there are indications of a similar pro-tumor function in hematological malignancy. Lee et al. demonstrated that monocytes have a promoting effect on migration and invasion of human B-cell precursor acute lymphoblastic leukemia (BCP-ALL) *in vitro* (Lee et al., 2012). Al-Matary et al. showed an increase of monocytes/macrophages in the BM of AML patients and AML mouse models, which might support the proliferation of AML cells *in vitro*, and it was also observed that the grade of macrophage infiltration was correlated with the survival of AML mice (Al-Matary et al., 2016). The proportion of monocytes and M0 macrophages in the high-risk group was significantly higher than in the low-risk group, which contributes to the poor prognosis of high-risk group patients. Tregs, which were regarded as suppressor T cells preventing autoimmunity, were found to be aberrantly accumulated in some types of tumor, playing a crucial role in dampening antitumor immunity and establishing an immunosuppressive microenvironment (Wang et al., 2017). Higher Treg percentages could indicate poor prognosis in a variety of cancer types (Overacre-Delgoffe and Vignali, 2018), and AML is no exception. Williams et al. found that an increased amount of Tregs in peripheral blood of AML patients is also associated with an increased risk of relapse (Williams et al., 2019). These studies are consistent with our findings that Treg proportion is higher in high-risk patients than low-risk patients. The detailed mechanism needs further investigation in the future. And the scRNA-seq transcriptome data can provide precision and details of the interaction between the tumor cells and the microenvironment. Zhang et al. developed a novel scRNA-seq data-based approach to reconstruct a multilayer signaling network that contains pathways from intercellular ligand–receptor interactions, intracellular transcriptional factors, and their target genes (Zhang et al., 2020). Meanwhile, the single-cell RNA-sequencing data based on multilayer network method (scMLnet) (Cheng et al., 2021) also help to resolve tumor–microenvironment interactions and dissect the microenvironment-mediated intercellular and intracellular signaling pathways of tumor cells, which might help to investigate the influence of microenvironment on the tumor growth, drug resistance, and patient prognosis.

Concerning treatment, Tregs have been targeted in the clinic, although the efficacy is limited (Overacre-Delgoffe and Vignali, 2018). Targeting macrophages with bisphosphonate could reduce angiogenesis and tumor growth in melanoma-bearing mice (Gazzaniga et al., 2007). In a mouse model of AML, Tregs accumulate at the site of disease and suppress the function of adoptively transferred cytotoxic T cells (CTL), and depletion of Tregs restored CTL function and reduced leukemia progression (Zhou et al., 2009). Anti-CD47 monoclonal antibody can inhibit the immune escape of AML leukemic stem cells to macrophages and play an antileukemic role by phagocytosis of leukemic stem cells through macrophages (Majeti et al., 2009). Clinical trials of CD47 mAb magrolimab (Hu5F9-G4) as a single agent or in combination for the treatment of relapsed refractory AML have shown promising efficacy (magrolimab, Phase I, ClinicalTrials.gov ID: NCT02678338; magrolimab + atezolizumab, Phase I, ClinicalTrials.gov ID: NCT03922477; magrolimab + azacitidine, Phase Ib, ClinicalTrials.gov ID: NCT03248479). Antiangiogenic therapy may be an effective method in AML patients. Reduced BM angiogenesis may help to restore drug sensitivity of drug-resistant AML (Lin et al., 2019). Antiangiogenic therapy may be an effective method in AML patients. Lin et al. demonstrated that wogonoside, one of the metabolites of traditional Chinese medicine Huangqin, could inhibit the BM angiogenesis and tumor progression of AML *in vivo* and *in vitro* (Lin et al., 2019). Based on the results of clinical trials, some antiangiogenic drugs that inhibit vascular endothelial growth factor (VEGF), such as bevacizumab (Karp et al., 2004), cediranib (Fiedler et al., 2010), AG-013736 (Giles et al., 2006), and SU5416 (Fiedler et al., 2003), could be an effective treatment for AML, either alone or in addition to chemotherapy that works independently on different targets. Hypoxia is becoming an emerging target in AML. Hypoxia-activated prodrug, a new class of anti-cancer agents, selectively deliver cytostatic or cytotoxic agents to hypoxic subregions, uncloak at low oxygen pressure, and release the active drug. Small-scale clinical trials about hypoxia-activated prodrugs PR-104 and TH-302 treating patients with relapsed and/or refractory AML were conducted, showing a definite antileukemia activity (Konopleva et al., 2015; Badar et al., 2016) (TH-302, Phase 1, clinicaltrials.gov ID: NCT01149915; PR-104, Phase 1, ClinicalTrials.gov ID: NCT01037556). BCL-2, a proapoptotic protein, could be overexpressed in hypoxia conditions; its inhibitors can reduce oxidative phosphorylation and eradicate quiescent chemo-resistant AML stem cells (Ashton et al., 2018). Echinomycin, a hypoxia-inducible factor HIF-1α inhibitor, can selectively kill the leukemia-initiating cell without affecting host HSCs in relapsed AML mice (Wang et al., 2014). The preliminary pathophysiological observation of this study may provide a perspective for further investigation and a potential therapeutic target in the future.

Although large cohorts were utilized to establish our model, there are still certain limitations in our study. The study was based on retrospective cohorts, lacking prospective cohorts, and experimental evidence. Data from different centers and various platforms are necessary to validate the performance of our model. Further studies including animal experiments and *in vitro* cellular experiments will be needed to confirm our findings and delineate the pathophysiological mechanism.

## Conclusion

In summary, our HPM, complex model, and nomogram had excellent predictive power for clinical applications, helping clinicians in making clinical decisions. Our hypoxia-related immune and metabolic alterations might help to find a potential therapeutic target.

## Data Availability

The original contributions presented in the study are included in the article material. The R Code used for this analysis is available as an additional file (Supplementary Data File 11). Further inquiries can be directed to the corresponding authors.
