# Supplementary figures and images for: Establishment of Prognosis Model in Acute Myeloid Leukemia Based on Hypoxia Microenvironment, and Exploration of Hypoxia-Related Mechanisms
Source: Front Genet. 2021 Oct 26;12:727392. doi: 10.3389/fgene.2021.727392 (PMC8578022; doi:10.3389/fgene.2021.727392)

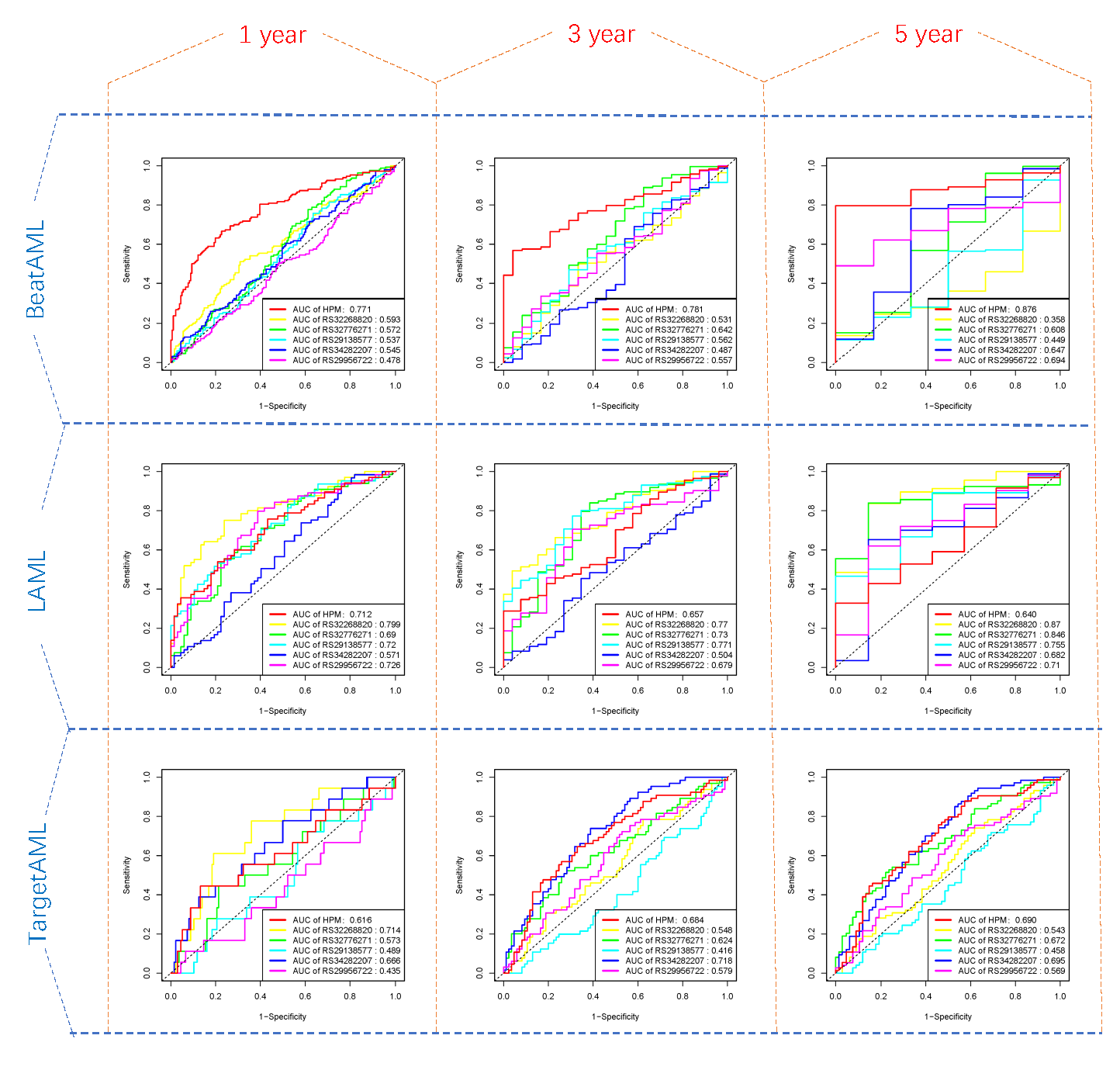

Supplement: Supplementary file 1 [file Image3.TIFF]

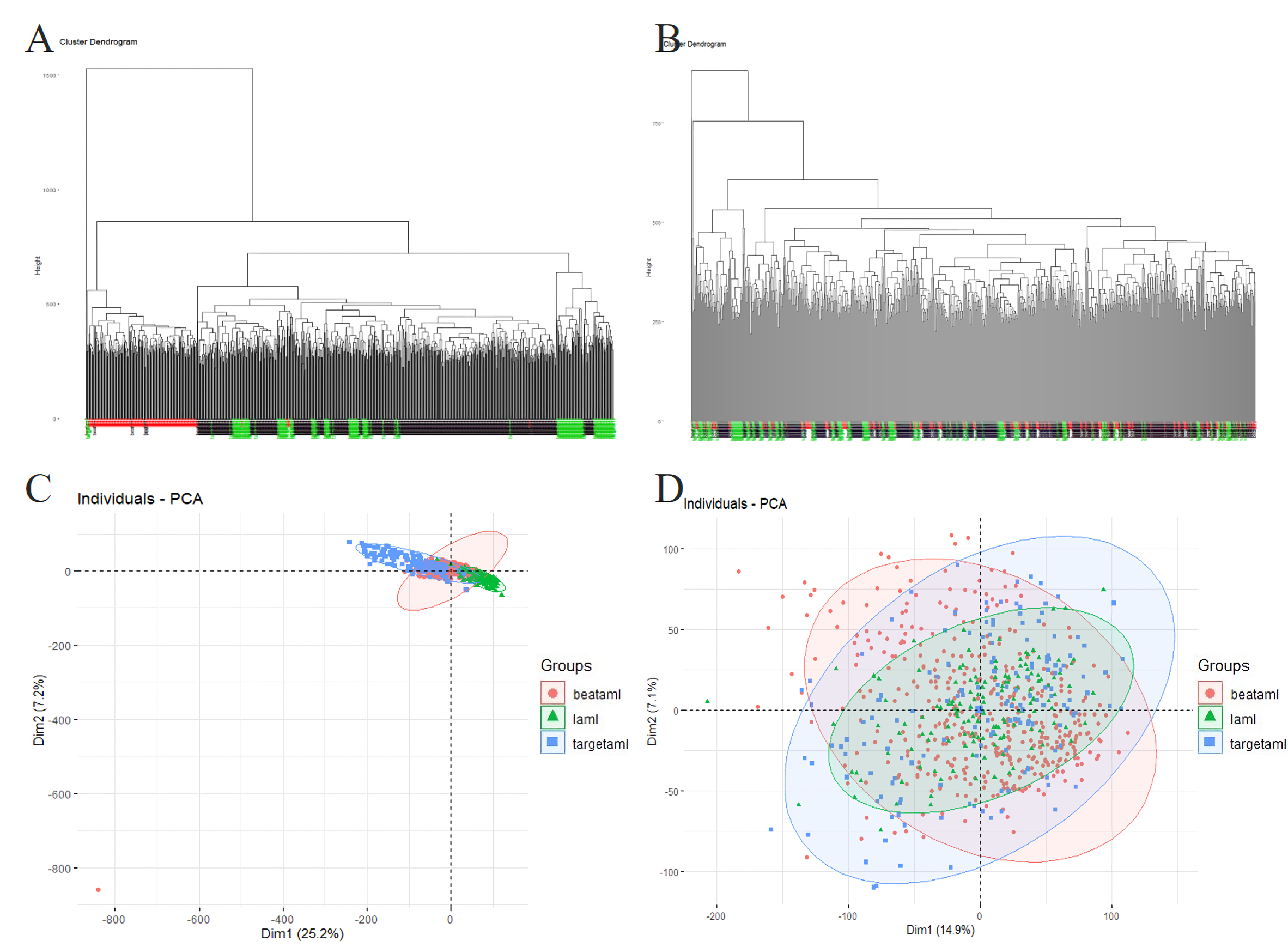

Supplement: Supplementary file 6 [file Image1.TIF]

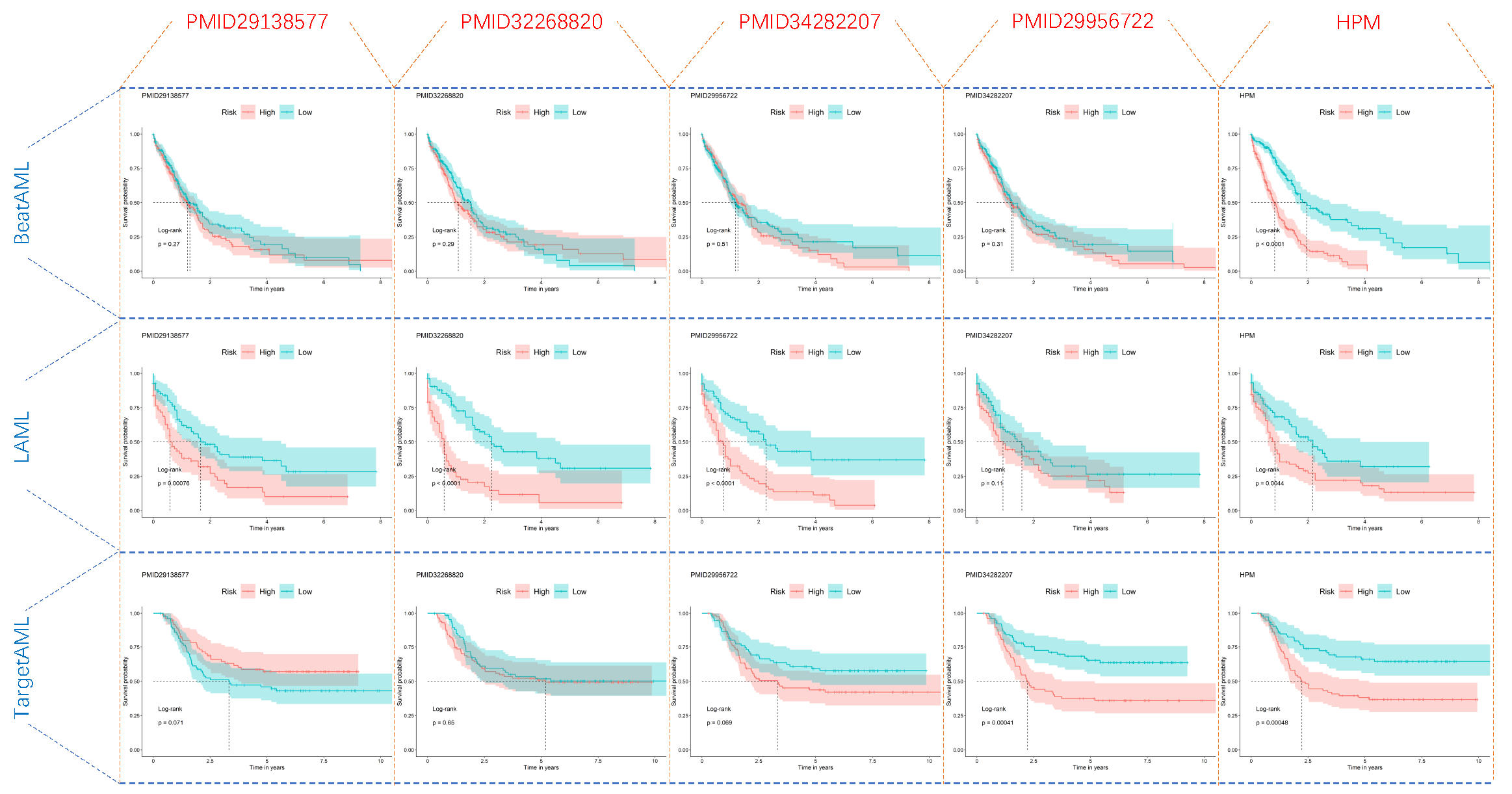

Supplement: Supplementary file 13 [file Image2.TIFF]
