# Supplementary material for: Establishment of Prognosis Model in Acute Myeloid Leukemia Based on Hypoxia Microenvironment, and Exploration of Hypoxia-Related Mechanisms
Source: Front Genet. 2021 Oct 26;12:727392. doi: 10.3389/fgene.2021.727392 (PMC8578022; doi:10.3389/fgene.2021.727392)
Supplement: Supplementary file 8 [file Table2.DOCX]

|  | Year(s) | PMID29138577 | PMID32268820 | PMID29956722 | PMID34282207 | HPM |
| --- | --- | --- | --- | --- | --- | --- |
| LAML | 1 | 0.72 | 0.799 | 0.726 | 0.571 | 0.712 |
|  | 3 | 0.771 | 0.77 | 0.679 | 0.504 | 0.657 |
|  | 5 | 0.755 | 0.87 | 0.71 | 0.682 | 0.64 |
| TargetAML | 1 | 0.489 | 0.714 | 0.435 | 0.666 | 0.616 |
|  | 3 | 0.416 | 0.548 | 0.579 | 0.718 | 0.684 |
|  | 5 | 0.458 | 0.543 | 0.569 | 0.695 | 0.69 |
| BeatAML | 1 | 0.537 | 0.593 | 0.478 | 0.545 | 0.711 |
|  | 3 | 0.562 | 0.531 | 0.557 | 0.487 | 0.781 |
|  | 5 | 0.449 | 0.358 | 0.694 | 0.647 | 0.876 |

Supplementary Table 2 The AUC values of the ROC analyses in various datasets using different predictive models.
