# Supplementary material for: Establishment of Prognosis Model in Acute Myeloid Leukemia Based on Hypoxia Microenvironment, and Exploration of Hypoxia-Related Mechanisms
Source: Front Genet. 2021 Oct 26;12:727392. doi: 10.3389/fgene.2021.727392 (PMC8578022; doi:10.3389/fgene.2021.727392)
Supplement: Supplementary file 14 [file DataSheet11.DOCX]

rm(list=ls())

#Model Establishment

##Univariate analysis

library(survival)

load(file = "ce_beataml_lhg.Rdata")

rt <- ce_beataml_lhg[,-1]

rt <- cbind(rt[,c('days', 'vital_status')], rt[,-which(names(rt)%in%c('days', 'vital_status'))])

rt$vital_status <- ifelse(rt$vital_status == 'Dead', 1, 0)

rt <- rt[rt$days>0,]

outTab = data.frame()

for(i in colnames(rt[,3:ncol(rt)])){

cox <- coxph(Surv(days, vital_status) ~ rt[,i], data = rt)

coxSummary = summary(cox)

outTab=rbind(outTab,cbind(gene=i,HR=coxSummary$coefficients[,"exp(coef)"],

z=coxSummary$coefficients[,"z"],

pvalue=coxSummary$coefficients[,"Pr(>|z|)"],

lower=coxSummary$conf.int[,'lower .95'],

upper=coxSummary$conf.int[,'upper .95']))

}

write.table(outTab,file="./step3_create_model/univariateCox.xls",sep="\t",row.names=F,quote=F)

tce_beataml_lhg <- t(ce_beataml_lhg)

write.table(tce_beataml_lhg,'./step3_create_model/tce_beataml_lhg.txt',sep = '\t',quote = F,col.names = F)

tce_beataml_lhg <- read.table('./step3_create_model/tce_beataml_lhg.txt', sep = '\t', row.names = 1, check.names = F)

n <- ncol(tce_beataml_lhg)

set.seed(2088)

beataml_train_index <- sample(1:n, 0.7*n)

beataml_trainset <- tce_beataml_lhg[,beataml_train_index]

beataml_testset <- tce_beataml_lhg[,-beataml_train_index]

write.table(beataml_train_index, './step3_create_model/set.seed.txt', sep = '\t')

write.table(beataml_trainset, './step3_create_model/beataml_trainset.txt', sep = '\t', col.names = F)

write.table(beataml_testset, './step3_create_model/beataml_testset.txt', sep = '\t', col.names = F)

LassoGenes <- read.table('./step3_create_model/univariateCox.xls', header = T, sep = '\t', check.names = F)

LassoGenes <- LassoGenes[LassoGenes$pvalue < 0.05,]

LassoGenes <- LassoGenes[,'gene']

LassoGenes <- data.frame(LassoGenes)

names(LassoGenes) <- 'id'

id <- c('days', 'vital_status')

id <- data.frame(id)

data1 <- rbind(id, LassoGenes)

data2 <- read.table('./step3_create_model/beataml_trainset.txt', header = T, sep = '\t', check.names = F)

data <- merge(data1, data2, by.x = 'id', by.y = 'id')

rt <- t(data)

write.table(rt, './step3_create_model/LassoCoxInput.txt', sep = '\t', col.names = F)

## lasso

library("glmnet")

library("survival")

rt <- read.table("./step3_create_model/LassoCoxInput.txt", header = T, sep = "\t", row.names = 1, check.names = F)

rt <- cbind(rt[,c('days', 'vital_status')], rt[,-which(names(rt)%in%c('days', 'vital_status'))])

rt$vital_status <- ifelse(rt$vital_status == 'Dead', 1, 0)

rt <- rt[rt$days>0,]

rt[,"days"]=rt[,"days"]/365

x <- as.matrix(rt[,c(3:ncol(rt))])

y <- data.matrix(Surv(rt$days, rt$vital_status))

fit <- glmnet(x, y, family = "cox", maxit = 1000000)

summary(fit)

pdf("./step3_create_model/lambda.pdf")

plot(fit, xvar = "lambda", label = TRUE)

dev.off()

cvfit <- cv.glmnet(x, y, family = "cox", maxit = 1000000)

cvfit$lambda.min

cvfit$lambda.1se

pdf("./step3_create_model/cvfit.pdf")

plot(cvfit)

abline(v = log(c(cvfit$lambda.min, cvfit$lambda.1se)), lty = "dashed")

dev.off()

coef <- coef(fit, s = cvfit$lambda.min)

index <- which(coef != 0)

actCoef <- coef[index]

lassoGene <- row.names(coef)[index]

write.table(lassoGene,file="./step3_create_model/lasso.txt",sep="\t",quote=F,row.names=F,col.names=F)

## multivariate analysis

MultiGenes <- read.table('./step3_create_model/lasso.txt', sep = '\t', check.names = F)

names(MultiGenes) <- 'id'

id <- c('days', 'vital_status')

id <- data.frame(id)

data1 <- rbind(id, MultiGenes)

data2 <- read.table('./step3_create_model/beataml_trainset.txt', header = T, sep = '\t', check.names = F)

data <- merge(data1, data2, by.x = 'id', by.y = 'id')

rt <- t(data)

write.table(rt, './step3_create_model/MultiCoxInput.txt', sep = '\t', col.names = F)

library(survival)

library(survminer)

rt <- read.table("./step3_create_model/MultiCoxInput.txt", header = T, sep = "\t", row.names = 1, check.names = F)

rt <- cbind(rt[,c('days', 'vital_status')], rt[,-which(names(rt)%in%c('days', 'vital_status'))])

rt$vital_status <- ifelse(rt$vital_status == 'Dead', 1, 0)

rt[,"days"]=rt[,"days"]/365

cox <- coxph(Surv(days, vital_status) ~ ., data = rt)

cox <- step(cox,direction = "both")

summary(cox)

riskScore <- predict(cox,type="risk",newdata=rt)

summary <- summary(cox)

coxGene <- rownames(summary$coefficients)

coxGene <- gsub("`","",coxGene)

outCol <- c("days","vital_status",coxGene)

risk <- as.vector(ifelse(riskScore>median(riskScore),"high","low"))

write.table(cbind(id=rownames(cbind(rt[,outCol],riskScore,risk)),cbind(rt[,outCol],riskScore,risk)),

file="./step3_create_model/beataml_train_risk.txt",

sep="\t",

quote=F,

row.names=F)

write.table(cbind(id=coxGene,summary$coefficients,summary$conf.int),

file="./step3_create_model/coxResult.xls",

sep="\t",

quote=F,

row.names=F)

cox

pdf('./step3_create_model/forest.pdf',width = 6,height = 4)

ggforest(cox,

main = "Hazard ratio",

cpositions = c(0.02,0.22, 0.4),

fontsize = 0.6,

refLabel = "reference",

noDigits = 2)

dev.off()

#test

data2 <- read.table('./step3_create_model/beataml_testset.txt', header = T, sep = '\t', check.names = F)

data <- merge(data1,data2,by.x = 'id',by.y = 'id')

rt <- t(data)

write.table(rt, './step3_create_model/MultiCoxInput-test.txt', sep = '\t', col.names = F)

rttest=read.table("./step3_create_model/MultiCoxInput-test.txt",header=T,sep="\t",check.names=F,row.names=1)

rttest <- cbind(rttest[,c('days', 'vital_status')], rttest[,-which(names(rttest)%in%c('days', 'vital_status'))])

rttest$vital_status <- ifelse(rttest$vital_status == 'Dead', 1, 0)

rttest[,"days"]=rttest[,"days"]/365

riskScoretest=predict(cox,type="risk",newdata=rttest)

summary=summary(cox)

coxGene=rownames(summary$coefficients)

coxGene=gsub("`","",coxGene)

outCol=c("days","vital_status",coxGene)

risktest=as.vector(ifelse(riskScoretest>median(riskScore),"high","low"))

write.table(cbind(id=rownames(cbind(rttest[,outCol],riskScoretest,risktest)),cbind(rttest[,outCol],riskScoretest,risktest)),

file="./step3_create_model/beataml_test_risk.txt",

sep="\t",

quote=F,

row.names=F)

#ROC

#training

library(survivalROC)

rt <- read.table("./step3_create_model/beataml_train_risk.txt",header=T,sep="\t",check.names=F,row.names=1)

pdf(file="./step3_create_model/roc_beataml_train.pdf",width = 6, height =6)

par(oma=c(0.5,1,0,1),font.lab=1.5,font.axis=1.5)

roc1=survivalROC(Stime=rt$days, status=rt$vital_status, marker = rt$riskScore,

predict.time =1, method="KM")

roc3=survivalROC(Stime=rt$days, status=rt$vital_status, marker = rt$riskScore,

predict.time =3, method="KM")

roc5=survivalROC(Stime=rt$days, status=rt$vital_status, marker = rt$riskScore,

predict.time =5, method="KM")

plot(roc1$FP, roc1$TP,type="l", xlim=c(0,1), ylim=c(0,1),col='green',

xlab="False positive rate", ylab="True positive rate",

main=paste("ROC curves of the training cohort"),

lwd = 2, cex.main=1.3, cex.lab=1.2, cex.axis=1.2, font=1.2)

legend('bottomright', c(paste("1-year AUC = ", round(roc1$AUC,3)),paste("3-year AUC = ", round(roc3$AUC,3)), paste("5-year AUC = ", round(roc5$AUC,3))),

cex=0.8, col=c("green","blue","red"), lwd = 2)

par(new=TRUE)

plot(roc3$FP, roc3$TP, type="l", col='blue',

xlab="", ylab="",axes = FALSE,lwd = 2, cex.main=1.3, cex.lab=1.2, cex.axis=1.2, font=1.2)

par(new=TRUE)

plot(roc5$FP, roc5$TP, type="l", col='red',

xlab="", ylab="",axes = FALSE,lwd = 2, cex.main=1.3, cex.lab=1.2, cex.axis=1.2, font=1.2)

abline(0,1)

dev.off()

#test

library(survivalROC)

rttest=read.table("./step3_create_model/beataml_test_risk.txt",header=T,sep="\t",check.names=F,row.names=1)

pdf(file="./step3_create_model/roc_beataml_test.pdf",width = 6, height =6)

par(oma=c(0.5,1,0,1),font.lab=1.5,font.axis=1.5)

roctest1=survivalROC(Stime=rttest$days, status=rttest$vital_status, marker = rttest$riskScoretest,

predict.time =1, method="KM")

roctest3=survivalROC(Stime=rttest$days, status=rttest$vital_status, marker = rttest$riskScoretest,

predict.time =3, method="KM")

roctest5=survivalROC(Stime=rttest$days, status=rttest$vital_status, marker = rttest$riskScoretest,

predict.time =5, method="KM")

plot(roctest1$FP, roctest1$TP,type="l", xlim=c(0,1), ylim=c(0,1),col='green',

xlab="False positive rate", ylab="True positive rate",

main=paste("ROC curves of the test cohort"),

lwd = 2, cex.main=1.3, cex.lab=1.2, cex.axis=1.2, font=1.2)

legend('bottomright', c(paste("1-year AUC = ", round(roctest1$AUC,3)),paste("3-year AUC = ", round(roctest3$AUC,3)), paste("5-year AUC = ", round(roctest5$AUC,3))),

cex=0.8, col=c("green","blue","red"), lwd = 2)

par(new=TRUE)

plot(roctest3$FP, roctest3$TP, type="l", col='blue',

xlab="", ylab="",axes = FALSE,lwd = 2, cex.main=1.3, cex.lab=1.2, cex.axis=1.2, font=1.2)

par(new=TRUE)

plot(roctest5$FP, roctest5$TP, type="l", col='red',

xlab="", ylab="",axes = FALSE,lwd = 2, cex.main=1.3, cex.lab=1.2, cex.axis=1.2, font=1.2)

abline(0,1)

dev.off()

library(survival)

library(rms)

nomtest=read.table("./step3_create_model/MultiCoxInput.txt",header=T,sep="\t",check.names=F,row.names=1,stringsAsFactors=F)

nomtest <- cbind(nomtest[,c('days', 'vital_status')], nomtest[,-which(names(nomtest)%in%c('days', 'vital_status'))])

nomtest$vital_status <- ifelse(nomtest$vital_status == 'Dead', 1, 0)

nomtest[,"days"]=nomtest[,"days"]/365

ddist <- datadist(nomtest)

options(datadist='ddist')

cox2 <- cph(Surv(days, vital_status) ~ .,surv=T,x=T, y=T,data=nomtest)

cox2 <- step(cox2,direction = "both")

surv <- Survival(cox2)

sur_1_year<-function(x)surv(1,lp=x)

sur_3_year<-function(x)surv(3,lp=x)

sur_5_year<-function(x)surv(5,lp=x)

nom_sur<- nomogram(cox2,fun=list(sur_1_year,sur_3_year,sur_5_year),lp= F,

funlabel=c('1-Year Survival','3-Year Survival','5-Year survival'),

maxscale=10,

fun.at=c('0.9','0.8','0.7','0.6','0.5','0.4','0.3','0.2','0.1'))

pdf("./step3_create_model/nom.pdf",13,8)

plot(nom_sur)

dev.off()

nom_sur

# Calibration curve

library(survival)

library(rms)

caltest=read.table("./step3_create_model/MultiCoxInput.txt",header=T,sep="\t",check.names=F,row.names=1,stringsAsFactors=F)

caltest <- cbind(caltest[,c('days', 'vital_status')], caltest[,-which(names(caltest)%in%c('days', 'vital_status'))])

caltest$vital_status <- ifelse(caltest$vital_status == 'Dead', 1, 0)

caltest[,"days"]=caltest[,"days"]/365

ddist <- datadist(caltest)

options(datadist='ddist')

units(caltest$days) <- "Year"

#calibration

cox1 <- cph(Surv(days,vital_status) ~ .,surv=T,x=T, y=T,time.inc = 1,data=caltest)

cox1 <- step(cox1,direction = "both")

cal1 <- calibrate(cox1, cmethod="KM", method="boot", u=1, m= 97, B=1000)

pdf("./step3_create_model/calibrate1.pdf")

plot(cal1,lwd=2,lty=1,errbar.col="gray",xlim = c(0,1),ylim = c(0,1),xlab ="Predicted Probability of 1-Year Survival",ylab="Actual 1-Year Survival",col="blue",sub=F)

mtext("")

box(lwd = 0.5)

abline(0,1,lty = 1,lwd = 2,col = "#E1C235")

lines(cal1[,c("mean.predicted","KM")],type="b",lwd=2,

col="blue", pch=16)

dev.off()

cox3 <- cph(Surv(days,vital_status) ~ .,surv=T,x=T, y=T,time.inc = 3,data=caltest)

cox3 <- step(cox3,direction = "both")

cal3 <- calibrate(cox3, cmethod="KM", method="boot", u=3, m= 97, B=1000)

plot(cal3,lwd=2,lty=1,errbar.col="gray",xlim = c(0,1),ylim = c(0,1),xlab ="Predicted Probability of 3-Year Survival",ylab="Actual 3-Year Survival",col="blue",sub=F)

mtext("")

box(lwd = 0.5)

abline(0,1,lty = 1,lwd = 2,col = "#E1C235")

lines(cal3[,c("mean.predicted","KM")],type="b",lwd=2,

col="blue", pch=16)

dev.off()

#5-year

cox5 <- cph(Surv(days,vital_status) ~ .,surv=T,x=T, y=T,time.inc = 5,data=caltest)

cox5 <- step(cox5,direction = "both")

cal5 <- calibrate(cox5, cmethod="KM", method="boot", u=5, m=97, B=1000)

pdf("./step3_create_model/calibrate5.pdf")

plot(cal5,lwd=2,lty=1,errbar.col="gray",xlim = c(0,1),ylim = c(0,1),xlab ="Predicted Probability of 5-Year Survival",ylab="Actual 5-Year Survival",col="blue",sub=F)

mtext("")

box(lwd = 0.5)

abline(0,1,lty = 1,lwd = 2,col = "#E1C235")

lines(cal5[,c("mean.predicted","KM")],type="b",lwd=2,

col="blue", pch=16)

dev.off()

#ROC curves

library(survival)

library(timeROC)

#train

roc2rt<-read.table("./step3_create_model/beataml_train_risk.txt",header=T,sep="\t")

roc2rt <- roc2rt[,c("id","days","vital_status","riskScore","risk")]

predict_1_year<- 1

predict_3_year<- 3

predict_5_year<- 5

ROC<-timeROC(T=roc2rt$days,delta=roc2rt$vital_status,

marker=roc2rt$riskScore,cause=1,

weighting="marginal",

times=c(predict_1_year,predict_3_year,predict_5_year),ROC=TRUE)

pdf("./step3_create_model/roc2_beataml_train.pdf")

plot(ROC,time=predict_1_year,col="green",title=FALSE,lwd=3)

plot(ROC,time=predict_3_year,col="blue",add=TRUE,title=FALSE,lwd=3)

plot(ROC,time=predict_5_year,col="red",add=TRUE,title=FALSE,lwd=3)

grid(nx = 5, ny = 5, col = "lightgray", lty = "dotted",

lwd = par("lwd"), equilogs = TRUE)

legend("bottomright",

c(paste("AUC of 1 year survival: ",round(ROC$AUC[1],3)),

paste("AUC of 3 year survival: ",round(ROC$AUC[2],3)),

paste("AUC of 5 year survival: ",round(ROC$AUC[3],3))),col=c("green","blue","red"),lwd=3)

dev.off()

#test

roc2rt<-read.table("./step3_create_model/beataml_test_risk.txt",header=T,sep="\t")

roc2rt <- roc2rt[,c("id","days","vital_status","riskScoretest")]

predict_1_year<- 1

predict_3_year<- 3

predict_5_year<- 5

ROC<-timeROC(T=roc2rt$days,delta=roc2rt$vital_status,

marker=roc2rt$riskScoretest,cause=1,

weighting="marginal",

times=c(predict_1_year,predict_3_year,predict_5_year),ROC=TRUE)

pdf("./step3_create_model/roc2_beataml_test.pdf")

plot(ROC,time=predict_1_year,col="green",title=FALSE,lwd=3)

plot(ROC,time=predict_3_year,col="blue",add=TRUE,title=FALSE,lwd=3)

plot(ROC,time=predict_5_year,col="red",add=TRUE,title=FALSE,lwd=3)

legend("bottomright",

c(paste("AUC of 1 year survival: ",round(ROC$AUC[1],3)),

paste("AUC of 3 year survival: ",round(ROC$AUC[2],3)),

paste("AUC of 5 year survival: ",round(ROC$AUC[3],3))),col=c("green","blue","red"),lwd=3)

dev.off()

#Kaplan-Meier survival analysis

# training

library(survminer)

library(survival)

surrt <- read.table("./step3_create_model/beataml_train_risk.txt",header=T,sep="\t")

risksurrt <- surrt[,c("days","vital_status","riskScore","risk")]

myfit <- survfit(Surv(days, vital_status) ~ risk, data = risksurrt)

surv_median(myfit)

p <- ggsurvplot(myfit, data = risksurrt,

surv.median.line = "hv",

legend.title = "risk",

legend.labs = c("High RiskScore", "Low RiskScore"),

pval = TRUE,

conf.int = TRUE,

palette = "jco",

ggtheme = theme_bw(),

ncensor.plot = TRUE,

risk.table = TRUE,

xlab = "Time in years",

ylab = "Survival probability")

ggsave(filename = "./step3_create_model/fig/beatmal_risksurvival.png",print(p))

#HR

# library(survival)

data.survdiff <- survdiff(Surv(days, vital_status) ~ risk, data = risksurrt)

p.val = 1 - pchisq(data.survdiff$chisq, length(data.survdiff$n) - 1)

HR = 1/((data.survdiff$obs[2]/data.survdiff$exp[2])/(data.survdiff$obs[1]/data.survdiff$exp[1]))

up95 = exp(log(HR) + qnorm(0.975)*sqrt(1/data.survdiff$exp[2]+1/data.survdiff$exp[1]))

low95 = exp(log(HR) - qnorm(0.975)*sqrt(1/data.survdiff$exp[2]+1/data.survdiff$exp[1]))

# test

library(survminer)

library(survival)

surrttest <- read.table("./step3_create_model/beataml_test_risk.txt",header=T,sep="\t")

risksurrttest <- surrttest[,c("days","vital_status","riskScoretest","risktest")]

myfit <- survfit(Surv(days, vital_status) ~ risktest, data = risksurrttest)

surv_median(myfit)

p <- ggsurvplot(myfit, data = risksurrttest,

surv.median.line = "hv",

legend.title = "risk",

legend.labs = c("High RiskScore", "Low RiskScore"),

pval = TRUE,

conf.int = TRUE,

palette = "jco",

ggtheme = theme_bw(),

ncensor.plot = TRUE,

risk.table = TRUE,

xlab = "Time in years",

ylab = "Survival probability")

ggsave(filename = "./step3_create_model/fig/risksurvivaltest.png",print(p))

#HR

data.survdiff.test <- survdiff(Surv(days, vital_status) ~ risktest, data = risksurrttest)

p.valtest = 1 - pchisq(data.survdiff.test$chisq, length(data.survdiff.test$n) - 1)

HRtest = 1/((data.survdiff.test$obs[2]/data.survdiff.test$exp[2])/(data.survdiff.test$obs[1]/data.survdiff.test$exp[1]))

up95test = exp(log(HRtest) + qnorm(0.975)*sqrt(1/data.survdiff.test$exp[2]+1/data.survdiff.test$exp[1]))

low95test = exp(log(HRtest) - qnorm(0.975)*sqrt(1/data.survdiff.test$exp[2]+1/data.survdiff.test$exp[1]))

#validation

#laml

load(file = "ce_laml_lhg.Rdata")

laml_clin_data <- ce_laml_lhg[,c("id","days","vital_status")]

write.table(laml_clin_data,'./clin/laml_clin/laml_clin_data.txt',sep = '\t',quote = F,col.names = T,row.names = F)

tce_laml_lhg <- t(ce_laml_lhg)

write.table(tce_laml_lhg,'./step4_external_validation/tce_laml_lhg.txt',sep = '\t',quote = F,col.names = F)

tce_laml_lhg <- read.table('./step4_external_validation/tce_laml_lhg.txt', header = T, sep = '\t', check.names = F)

data2 <- tce_laml_lhg

data <- merge(data1,data2,by.x = 'id',by.y = 'id')

rt <- t(data)

write.table(rt, './step4_external_validation/MultiCoxInput-laml.txt', sep = '\t', col.names = F)

rttest=read.table("./step4_external_validation/MultiCoxInput-laml.txt",header=T,sep="\t",check.names=F,row.names=1)

rttest <- cbind(rttest[,c('days', 'vital_status')], rttest[,-which(names(rttest)%in%c('days', 'vital_status'))])

rttest$vital_status <- ifelse(rttest$vital_status == 'Dead', 1, 0)

rttest[,"days"]=rttest[,"days"]/365

riskScoretest=predict(cox,type="risk",newdata=rttest)

summary=summary(cox)

coxGene=rownames(summary$coefficients)

coxGene=gsub("`","",coxGene)

outCol=c("days","vital_status",coxGene)

risktest=as.vector(ifelse(riskScoretest>median(riskScore),"high","low"))

write.table(cbind(id=rownames(cbind(rttest[,outCol],riskScoretest,risktest)),cbind(rttest[,outCol],riskScoretest,risktest)),

file="./step4_external_validation/laml_risk.txt",

sep="\t",

quote=F,

row.names=F)

#ROC

library(survivalROC)

rt <- read.table("./step4_external_validation/laml_risk.txt",header=T,sep="\t",check.names=F,row.names=1)

pdf(file="./step4_external_validation/roc_laml.pdf",width = 6, height =6)

par(oma=c(0.5,1,0,1),font.lab=1.5,font.axis=1.5)

roc1=survivalROC(Stime=rt$days, status=rt$vital_status, marker = rt$riskScoretest,

predict.time =1, method="KM")

roc3=survivalROC(Stime=rt$days, status=rt$vital_status, marker = rt$riskScoretest,

predict.time =3, method="KM")

roc5=survivalROC(Stime=rt$days, status=rt$vital_status, marker = rt$riskScoretest,

predict.time =5, method="KM")

plot(roc1$FP, roc1$TP,type="l", xlim=c(0,1), ylim=c(0,1),col='green',

xlab="False positive rate", ylab="True positive rate",

main=paste("ROC curves of the training cohort"),

lwd = 2, cex.main=1.3, cex.lab=1.2, cex.axis=1.2, font=1.2)

legend('bottomright', c(paste("1-year AUC = ", round(roc1$AUC,3)),paste("3-year AUC = ", round(roc3$AUC,3)), paste("5-year AUC = ", round(roc5$AUC,3))),

cex=0.8, col=c("green","blue","red"), lwd = 2)

par(new=TRUE)

plot(roc3$FP, roc3$TP, type="l", col='blue',

xlab="", ylab="",axes = FALSE,lwd = 2, cex.main=1.3, cex.lab=1.2, cex.axis=1.2, font=1.2)

par(new=TRUE)

plot(roc5$FP, roc5$TP, type="l", col='red',

xlab="", ylab="",axes = FALSE,lwd = 2, cex.main=1.3, cex.lab=1.2, cex.axis=1.2, font=1.2)

abline(0,1)

dev.off()

#targetaml

load(file = "ce_targetaml_lhg.Rdata")

tce_targetaml_lhg <- t(ce_targetaml_lhg)

write.table(tce_targetaml_lhg,'./step4_external_validation/tce_targetaml_lhg.txt',sep = '\t',quote = F,col.names = F)

tce_targetaml_lhg <- read.table('./step4_external_validation/tce_targetaml_lhg.txt', header = T, sep = '\t', check.names = F)

data2 <- tce_targetaml_lhg

data <- merge(data1,data2,by.x = 'id',by.y = 'id')

rt <- t(data)

write.table(rt, './step4_external_validation/MultiCoxInput-targetaml.txt', sep = '\t', col.names = F)

rttest=read.table("./step4_external_validation/MultiCoxInput-targetaml.txt",header=T,sep="\t",check.names=F,row.names=1)

rttest <- cbind(rttest[,c('days', 'vital_status')], rttest[,-which(names(rttest)%in%c('days', 'vital_status'))])

rttest$vital_status <- ifelse(rttest$vital_status == 'Dead', 1, 0)

rttest[,"days"]=rttest[,"days"]/365

riskScoretest=predict(cox,type="risk",newdata=rttest)

summary=summary(cox)

coxGene=rownames(summary$coefficients)

coxGene=gsub("`","",coxGene)

outCol=c("days","vital_status",coxGene)

risktest=as.vector(ifelse(riskScoretest>median(riskScore),"high","low"))

write.table(cbind(id=rownames(cbind(rttest[,outCol],riskScoretest,risktest)),cbind(rttest[,outCol],riskScoretest,risktest)),

file="./step4_external_validation/targetaml_risk.txt",

sep="\t",

quote=F,

row.names=F)

#ROC

library(survivalROC)

rt <- read.table("./step4_external_validation/targetaml_risk.txt",header=T,sep="\t",check.names=F,row.names=1)

pdf(file="./step4_external_validation/roc_targetaml.pdf",width = 6, height =6)

par(oma=c(0.5,1,0,1),font.lab=1.5,font.axis=1.5)

roc1=survivalROC(Stime=rt$days, status=rt$vital_status, marker = rt$riskScoretest,

predict.time =1, method="KM")

roc3=survivalROC(Stime=rt$days, status=rt$vital_status, marker = rt$riskScoretest,

predict.time =3, method="KM")

roc5=survivalROC(Stime=rt$days, status=rt$vital_status, marker = rt$riskScoretest,

predict.time =5, method="KM")

plot(roc1$FP, roc1$TP,type="l", xlim=c(0,1), ylim=c(0,1),col='green',

xlab="False positive rate", ylab="True positive rate",

main=paste("ROC curves of the training cohort"),

lwd = 2, cex.main=1.3, cex.lab=1.2, cex.axis=1.2, font=1.2)

legend('bottomright', c(paste("1-year AUC = ", round(roc1$AUC,3)),paste("3-year AUC = ", round(roc3$AUC,3)), paste("5-year AUC = ", round(roc5$AUC,3))),

cex=0.8, col=c("green","blue","red"), lwd = 2)

par(new=TRUE)

plot(roc3$FP, roc3$TP, type="l", col='blue',

xlab="", ylab="",axes = FALSE,lwd = 2, cex.main=1.3, cex.lab=1.2, cex.axis=1.2, font=1.2)

par(new=TRUE)

plot(roc5$FP, roc5$TP, type="l", col='red',

xlab="", ylab="",axes = FALSE,lwd = 2, cex.main=1.3, cex.lab=1.2, cex.axis=1.2, font=1.2)

abline(0,1)

dev.off()

#cibersort

rm(list = ls())

options(stringsAsFactors = F)

load(file = './step5_cibersort/input.Rdata')

Y[1:4,1:4]

X[1:4,1:4]

dim(X)

dim(Y)

library(preprocessCore)

library(parallel)

library(e1071)

source("./step5_cibersort/CIBERSORT.R")

sig_matrix = './step5_cibersort/LM22-ref.txt'

mixture_file = './step5_cibersort/beataml_log2_TPM_ciber.txt'

X <- read.table(sig_matrix,header=T,sep="\t",row.names=1,check.names=F)

Y <- read.table(mixture_file, header=T, sep="\t", check.names=F)

CIBERSORT(sig_matrix, mixture_file, perm=1000, QN=F)

#DEG

rm(list = ls())

mb_genelist <- read.csv("./step6_metabolism/metabolism_genelist.csv",head=T,sep=",")

mb_genelist <- mb_genelist[,1]

load("./step6_metabolism/beataml_counts_symbol.Rdata")

beataml_counts_mb <- beataml_counts[rownames(beataml_counts)%in%mb_genelist,]

risk_sur_beataml_all <- read.table("./step5_cibersort/risk_sco_sur_beataml_all.txt",header=T,sep="\t",check.names=F)

#str(beataml_train_risk)

table(risk_sur_beataml_all$id %in% colnames(beataml_counts_mb))

beataml_counts_mb <- beataml_counts_mb[,colnames(beataml_counts_mb) %in% risk_sur_beataml_all$id]

risk_sur_beataml_all=risk_sur_beataml_all[order(risk_sur_beataml_all$riskScore,decreasing = T),]

highlow25 <- risk_sur_beataml_all[c(1:105,315:419),]

head(highlow25)

exprSet <- beataml_counts_mb[,match(highlow25$id, colnames(beataml_counts_mb))]

save(exprSet, file = "./step6_metabolism/exprSet.Rdata")

group_list <- c(rep('high',105),rep('low',105))

#limma

suppressMessages(library(limma))

library(edgeR)

design <- model.matrix(~0+factor(group_list))

colnames(design)=levels(factor(group_list))

rownames(design)=colnames(exprSet)

design

Unnormalised <- DGEList(counts=exprSet)$counts

dge <- DGEList(counts=exprSet)

dge <- calcNormFactors(dge)

logCPM <- cpm(dge, log=TRUE, prior.count=3)

logCPM[1:4,1:4]

comp='high-low'

v <- voom(dge,design,plot=TRUE, normalize="quantile")

fit <- lmFit(v, design)

cont.matrix=makeContrasts(contrasts=c(comp),levels = design)

fit2=contrasts.fit(fit,cont.matrix)

fit2=eBayes(fit2)

save(fit2,file = "./step6_metabolism/limma_analysis_fit2.Rdata")

exprSet_new=v$E

par(cex = 0.7)

n.sample=ncol(exprSet)

if(n.sample>40) par(cex = 0.5)

cols <- rainbow(n.sample*1.2)

par(mfrow=c(2,2))

boxplot(Unnormalised, col = cols,main="Unnormalised expression value",las=2,cex.axis=0.4)

boxplot(exprSet_new, col = cols,main="voom normalised expression value",las=2,cex.axis=0.4)

hist(Unnormalised)

hist(exprSet_new)

dev.off()

tempOutput = topTable(fit2, coef=comp, n=Inf)

DEG_limma_voom = na.omit(tempOutput)

head(DEG_limma_voom)

nrDEG=DEG_limma_voom[,c(1,4)]

colnames(nrDEG)=c('log2FoldChange','pvalue')

save(nrDEG, DEG_limma_voom, file = "./step6_metabolism/limma.Rdata")

load("./step6_metabolism/limma.Rdata")

colnames(DEG_limma_voom)

nrDEG3 = DEG_limma_voom

## heatmap

nrDEG=nrDEG3

library(pheatmap)

choose_gene=head(rownames(nrDEG),50)

choose_matrix=exprSet[choose_gene,]

choose_matrix=t(scale(t(choose_matrix)))

pheatmap(choose_matrix,filename = './step6_metabolism/pheatmap.png')

dev.off()

#volcano

library(ggplot2)

DEG=nrDEG3

colnames(DEG)

plot(DEG$logFC,-log2(DEG$P.Value))

logFC_cutoff=1

DEG$change = as.factor(ifelse(DEG$P.Value < 0.05 & abs(DEG$logFC) > logFC_cutoff,

ifelse(DEG$logFC > logFC_cutoff ,'UP','DOWN'),'NOT')

)

table(DEG$change)

this_tile <- paste0('Cutoff for logFC is ',round(logFC_cutoff,3),

'\nThe number of up gene is ',nrow(DEG[DEG$change =='UP',]) ,

'\nThe number of down gene is ',nrow(DEG[DEG$change =='DOWN',])

)

g = ggplot(data=DEG, aes(x=logFC, y=-log10(P.Value),color=change)) + geom_point(alpha=0.4, size=1.75) +

theme_set(theme_set(theme_bw(base_size=20)))+ xlab("log2 fold change") + ylab("-log10 p-value") +

ggtitle( this_tile ) + theme(plot.title = element_text(size=15,hjust = 0.5)) +

scale_colour_manual(values = c('blue','black','red'))

print(g)

ggsave(g,filename = './step6_metabolism/volcano.png')

#KEGG & GO enrichment analysis

library(org.Hs.eg.db)

library(data.table)

library(clusterProfiler)

map_dt <- bitr(rownames(DEG), fromType = "SYMBOL",toType = c( "ENTREZID"),OrgDb = org.Hs.eg.db)

DEG$ENTREZID <- map_dt[match(rownames(DEG),map_dt$SYMBOL),2]

head(DEG)

{

gene_up = DEG[ DEG$change == 'UP', 'ENTREZID' ]

gene_down = DEG[ DEG$change == 'DOWN', 'ENTREZID' ]

gene_diff = c( gene_up, gene_down )

gene_all = as.character(DEG[ ,'ENTREZID'] )

}

{

geneList = DEG$logFC

names( geneList ) = DEG$ENTREZID

geneList = sort( geneList, decreasing = T )

}

{

## KEGG pathway analysis

kk.up <- enrichKEGG( gene = gene_up ,

organism = 'hsa' ,

universe = gene_all ,

pvalueCutoff = 0.05 ,

qvalueCutoff = 0.05 )

kk.down <- enrichKEGG( gene = gene_down ,

organism = 'hsa' ,

universe = gene_all ,

pvalueCutoff = 0.05 ,

qvalueCutoff = 0.05 )

}

library( ggplot2 )

{

kegg_down_dt <- as.data.frame( kk.down )

kegg_up_dt <- as.data.frame( kk.up )

down_kegg <- kegg_down_dt[ kegg_down_dt$pvalue < 0.05, ]

down_kegg$group = -1

up_kegg <- kegg_up_dt[ kegg_up_dt$pvalue < 0.05, ]

up_kegg$group = 1

dat = rbind( up_kegg, down_kegg )

dat$pvalue = -log10( dat$pvalue )

dat$pvalue = dat$pvalue * dat$group

dat = dat[ order( dat$pvalue, decreasing = F ), ]

g_kegg <- ggplot( dat,

aes(x = reorder( Description, order( pvalue, decreasing=F ) ), y = pvalue, fill = group)) +

geom_bar( stat = "identity" ) +

scale_fill_gradient( low = "blue", high = "red", guide = FALSE ) +

scale_x_discrete( name = "Pathway names" ) +

scale_y_continuous( name = "log10P-value" ) +

coord_flip() + theme_bw() + theme( plot.title = element_text( hjust = 0.5 ) ) +

ggtitle( "Pathway Enrichment" )

print( g_kegg )

ggsave( g_kegg, filename = './step6_metabolism/kegg_up_down_anno.png' )

}

library(AnnotationHub)

library(biomaRt)

# KEGG result visualization

enrichKKup=DOSE::setReadable(kk.up, OrgDb='org.Hs.eg.db',keyType='ENTREZID')

enrichKKdown=DOSE::setReadable(kk.down, OrgDb='org.Hs.eg.db',keyType='ENTREZID')

#barplot

barplot(kk.up, showCategory = 10)

ggsave("./step6_metabolism/kegg_up_barplot.png", plot = last_plot())

dev.off()

#dotplot

dotplot(kk.up, showCategory = 10)

ggsave("./step6_metabolism/kegg_up_dotplot.png", plot = last_plot())

dev.off()

#barplot

barplot(kk.down, showCategory = 10)

ggsave("./step6_metabolism/kegg_down_barplot.png", plot = last_plot())

dev.off()

#dotplot

dotplot(kk.down, showCategory = 10)

ggsave("./step6_metabolism/kegg_down_dotplot.png", plot = last_plot())

dev.off()

#pathway net

png("./step6_metabolism/kegg_up_pathway_net.png",res=100,width = 820,height = 770)

emapplot(kk.up, showCategory = 30)

dev.off()

pdf("./step6_metabolism/kegg_down_pathway_net.pdf",width = 10,height = 9.5)

emapplot(kk.down, showCategory = 30)

dev.off()

#heatplot

heatplot(enrichKKup)

png("./step6_metabolism/kegg_upgene_pathway_heatmap.png",res=100,width = 1442,height = 523)

heatplot(enrichKKup, foldChange=geneList)

dev.off()

png("./step6_metabolism/kegg_updown_pathway_heatmap.png",res=110,width = 628,height = 276)

heatplot(enrichKKdown, foldChange=geneList)

dev.off()

#upsetplot

png("./step6_metabolism/upsetplot(enrichKKup).png",res=100,width = 728,height = 576)

upsetplot(enrichKKup)

dev.off()

# GO database analysis

{

g_list=list(gene_up=gene_up,

gene_down=gene_down,

gene_diff=gene_diff)

if(T){

go_enrich_results <- lapply( g_list , function(gene) {

lapply( c('BP','MF','CC') , function(ont) {

cat(paste('Now process ',ont ))

ego <- enrichGO(gene = gene,

universe = gene_all,

OrgDb = org.Hs.eg.db,

ont = ont ,

pAdjustMethod = "BH",

pvalueCutoff = 0.05,

qvalueCutoff = 0.05,

readable = TRUE)

print( head(ego) )

return(ego)

})

})

save(go_enrich_results,file = './step6_metabolism/go_enrich_results.Rdata')

}

}

library(clusterProfiler)

library(ggplot2)

library(enrichplot)

library(cowplot)

library(stringr)

library(topGO)

library(Rgraphviz)

# GO result visualization

{

load(file = './step6_metabolism/go_enrich_results.Rdata')

n1= c('gene_up','gene_down','gene_diff')

n2= c('BP','MF','CC')

for (i in 1:3){

for (j in 1:3){

fn=paste0('./step6_metabolism/go_dotplot_',n1[i],'_',n2[j],'.png')

cat(paste0(fn,'\n'))

png(fn,res=150,width = 780,height = 780)

p <- dotplot(go_enrich_results[[i]][[j]])

print(p+scale_color_continuous(low='red' ,high='blue')+theme_bw()+scale_y_discrete(labels=function(y) str_wrap(y,width = 25)))

dev.off()

}

}

}

# compare with other models

t_e_beataml <- read.csv("./step2_sva_combat/e_beataml_cb.csv",row.names=1,header = T)

e_beataml <- as.data.frame(t(t_e_beataml))

e_beataml[1:10,1:10]

e_beataml$id <- rownames(e_beataml)

load(file = "./clin/beataml_clin/beataml_clin_data.Rdata")

c_beataml <- beataml_clin_data[,c("id","days","vital_status")]

c_beataml <- c_beataml[-which(c_beataml$id=="BA2063"),]#离群值BA2063

ce_beataml <- merge(e_beataml, c_beataml, by="id", all.x = F,all.y = T)

rownames(ce_beataml) <- ce_beataml$id

e_beataml <- ce_beataml[,-which(colnames(ce_beataml)%in%c("id","days","vital_status"))]

c_beataml <- ce_beataml[,which(colnames(ce_beataml)%in%c("id","days","vital_status"))]

e_beataml[1:10,1:10]

c_beataml[1:10,]

c_beataml$vital_status <- ifelse(c_beataml$vital_status=="Alive",0,1)

c_beataml_roc <- c_beataml

c_beataml_km <- c_beataml

genelist <- colnames(e_beataml)

suit<-function(their_gene_list){

sum(their_gene_list %in% genelist)==length(their_gene_list)

}

#other

PMID32268820 <- c("TET3","CLEC11A","ATOX1","S100A4","BATF","PTP4A3","SPATS2L","SDHA")

suit(PMID32268820)

c_beataml_roc$RS32268820 = (-0.40986964)*e_beataml[,"TET3"]+0.424257154*e_beataml[,"S100A4"]+0.371192948*e_beataml[,"BATF"]+(0.321988462)*e_beataml[,"CLEC11A"]+0.500628619*e_beataml[,"PTP4A3"]+0.186937132*e_beataml[,"SPATS2L"]+0.255904972*e_beataml[,"SDHA"]+(-0.240853413)*e_beataml[,"ATOX1"]

c_beataml_km$GP32268820 <- ifelse(c_beataml_roc$RS32268820>=median(c_beataml_roc$RS32268820),'high','low')

PMID29138577 <- c("ACSF2","CXCR6","FAM124B","FRYL","GYPA","HBG1","MAGOH","MDH2","SLC2A5","SUCLG1","TMC5")

suit(PMID29138577)

c_beataml_roc$RS29138577 <- e_beataml[,"ACSF2"]*(0.438)+e_beataml[,"CXCR6"]*(-0.077)+e_beataml[,"FAM124B"]*(0.295)+e_beataml[,"FRYL"]*(-0.391)+e_beataml[,"GYPA"]*(-0.289)+e_beataml[,"HBG1"]*(-0.072)+e_beataml[,"MAGOH"]*(0.387)+e_beataml[,"MDH2"]*(-0.106)+e_beataml[,"SLC2A5"]*(0.329)+e_beataml[,"SUCLG1"]*(0.221)+e_beataml[,"TMC5"]*(-0.35)

c_beataml_km$GP29138577 <- ifelse(c_beataml_roc$RS29138577>=median(c_beataml_roc$RS29138577),'high','low')

PMID34282207 <- c("ENO3", "F3", "CCNA2" , "SLC2A5")

suit(PMID34282207)

c_beataml_roc$RS34282207 <- 0.81*e_beataml[,"ENO3"]+0.55*e_beataml[,"F3"]+0.41*e_beataml[,"CCNA2"]+0.47*e_beataml[,"SLC2A5"]

c_beataml_km$GP34282207 <- ifelse(c_beataml_roc$RS34282207>=median(c_beataml_roc$RS34282207),'high','low')

PMID29956722 <- c("TREML2","SLC7A11","NLRP2","DDIT4","LSP1","CLEC11A")

suit(PMID29956722)

c_beataml_roc$RS29956722 <- 1.053*e_beataml[,"TREML2"]+0.426*e_beataml[,"SLC7A11"]+0.222*e_beataml[,"NLRP2"]+0.548*e_beataml[,"DDIT4"]+(-0.771)*e_beataml[,"LSP1"]+(-0.396)*e_beataml[,"CLEC11A"]

c_beataml_km$GP29956722 <- ifelse(c_beataml_roc$RS29956722>=median(c_beataml_roc$RS29956722),'high','low')

#===========ROC curves=========================

library(timeROC)

library(survival)

rocCol=rainbow(ncol(c_beataml_roc)-2)

pdf(file="./step8_compare/diffModel_ROC_BeatAML_1.pdf",width=6,height=6)

j=1

aucText=c()

timeAUC=c()

addplot <- c(F,rep(T,ncol(c_beataml_roc)-4))

for(i in colnames(c_beataml_roc[,4:ncol(c_beataml_roc)])){

ROC<-timeROC(T=c_beataml_roc$days,delta=c_beataml_roc$vital_status,

marker=as.numeric(c_beataml_roc[,i]),cause=1,

weighting="marginal",

times=1*365,ROC=TRUE)

plot(ROC,time=1*365,col=rocCol[j+1],title=FALSE,lwd=2,add=addplot[j])

j=j+1

timeAUC=c(timeAUC,round(ROC$AUC[2],3))

}

roc <- timeROC(T=clindata$days,delta=clindata$vital_status,

marker=clindata$riskScore,cause=1,

weighting="marginal",

times=1,ROC=TRUE)

lines(roc$FP, roc$TP, type="l", xlim=c(0,1), ylim=c(0,1),col=rocCol[1],

xlab="False positive rate", ylab="True positive rate",

lwd = 2, cex.main=1.3, cex.lab=1.2, cex.axis=1.2, font=1.2)

textAUC <- c()

for (k in 1:(ncol(c_beataml_roc)-3)) {

textAUC=c(textAUC,paste("AUC of", colnames(c_beataml_roc)[k+3],":",round(timeAUC[k],3)))}

textAUC=c(paste0("HPM"," (AUC=",sprintf("%.3f",roc$AUC[2]),")"),textAUC)

legend("bottomright",textAUC, col=rocCol,lwd=3)

dev.off()

pdf(file="./step8_compare/diffModel_ROC_BeatAML_3.pdf",width=6,height=6)

j=1

aucText=c()

timeAUC=c()

addplot <- c(F,rep(T,ncol(c_beataml_roc)-4))

for(i in colnames(c_beataml_roc[,4:ncol(c_beataml_roc)])){

ROC<-timeROC(T=c_beataml_roc$days,delta=c_beataml_roc$vital_status,

marker=as.numeric(c_beataml_roc[,i]),cause=1,

weighting="marginal",

times=3*365,ROC=TRUE)

plot(ROC,time=3*365,col=rocCol[j+1],title=FALSE,lwd=2,add=addplot[j])

j=j+1

timeAUC=c(timeAUC,round(ROC$AUC[2],3))

}

roc <- timeROC(T=clindata$days,delta=clindata$vital_status,

marker=clindata$riskScore,cause=1,

weighting="marginal",

times=3,ROC=TRUE)

lines(roc$FP, roc$TP, type="l", xlim=c(0,1), ylim=c(0,1),col=rocCol[1],

xlab="False positive rate", ylab="True positive rate",

lwd = 2, cex.main=1.3, cex.lab=1.2, cex.axis=1.2, font=1.2)

textAUC <- c()

for (k in 1:(ncol(c_beataml_roc)-3)) {

textAUC=c(textAUC,paste("AUC of", colnames(c_beataml_roc)[k+3],":",round(timeAUC[k],3)))}

textAUC=c(paste0("HPM"," (AUC=",sprintf("%.3f",roc$AUC[2]),")"),textAUC)

legend("bottomright",textAUC, col=rocCol,lwd=3)

dev.off()

pdf(file="./step8_compare/diffModel_ROC_BeatAML_5.pdf",width=6,height=6)

j=1

aucText=c()

timeAUC=c()

addplot <- c(F,rep(T,ncol(c_beataml_roc)-4))

for(i in colnames(c_beataml_roc[,4:ncol(c_beataml_roc)])){

ROC<-timeROC(T=c_beataml_roc$days,delta=c_beataml_roc$vital_status,

marker=as.numeric(c_beataml_roc[,i]),cause=1,

weighting="marginal",

times=5*365,ROC=TRUE)

plot(ROC,time=5*365,col=rocCol[j+1],title=FALSE,lwd=2,add=addplot[j])

j=j+1

timeAUC=c(timeAUC,round(ROC$AUC[2],3))

}

roc <- timeROC(T=clindata$days,delta=clindata$vital_status,

marker=clindata$riskScore,cause=1,

weighting="marginal",

times=5,ROC=TRUE)

lines(roc$FP, roc$TP, type="l", xlim=c(0,1), ylim=c(0,1),col=rocCol[1],

xlab="False positive rate", ylab="True positive rate",

lwd = 2, cex.main=1.3, cex.lab=1.2, cex.axis=1.2, font=1.2)

textAUC <- c()

for (k in 1:(ncol(c_beataml_roc)-3)) {

textAUC=c(textAUC,paste("AUC of", colnames(c_beataml_roc)[k+3],":",round(timeAUC[k],3)))}

textAUC=c(paste0("HPM"," (AUC=",sprintf("%.3f",roc$AUC[2]),")"),textAUC)

legend("bottomright",textAUC, col=rocCol,lwd=3)

dev.off()

#========= recommended forvisualizing survival curves

library(survival)

library(survminer)

library(ggplot2)

library(stringr)

for (i in 4:ncol(c_beataml_km)) {

modelname <- colnames(c_beataml_km)[4:ncol(c_beataml_km)]

name <- colnames(c_beataml_km)[i]

group <- c_beataml_km[,i]

survival_dat <- data.frame(group = group,

status = c_beataml_km$vital_status,

time = c_beataml_km$days/365,

stringsAsFactors = F)

fit <- survfit(Surv(time, status) ~ group, data = survival_dat)

ggsurvplot(fit, data = survival_dat,

surv.median.line = "hv",

legend.title = "Risk",

title=paste("PMID",str_sub(name,3,10),sep = ''),

legend.labs = c("High","Low"),

pval = TRUE,

pval.method=TRUE,

conf.int = TRUE,

xlab = "Time in years",

ylab = "Survival probability")

last_plot() + theme(legend.key.size = unit(0.5, "inches"),

legend.title=element_text(size=18),

legend.text=element_text(size=18))

ggsave(filename = paste('./step8_compare/', "BeatAML-", name, '.png', sep = ''),plot = last_plot(),wi=8.97,hei=6.69)

}

fit1 <- survfit(Surv(days, vital_status) ~ risk, data = clindata)

ggsurvplot(fit1, data = clindata,

surv.median.line = "hv",

legend.title = "Risk",

title=paste("HPM"),

legend.labs = c("High","Low"),

pval = TRUE,

pval.method=TRUE,

conf.int = TRUE,

xlab = "Time in years",

ylab = "Survival probability")

last_plot() + theme(legend.key.size = unit(0.5, "inches"),

legend.title=element_text(size=18),

legend.text=element_text(size=18))

ggsave("./step8_compare/BeatAML-HPM.png", plot = last_plot(),wi=8.97,hei=6.69)
