# Supplementary material for: Establishment of Prognosis Model in Acute Myeloid Leukemia Based on Hypoxia Microenvironment, and Exploration of Hypoxia-Related Mechanisms
Source: Front Genet. 2021 Oct 26;12:727392. doi: 10.3389/fgene.2021.727392 (PMC8578022; doi:10.3389/fgene.2021.727392)
Supplement: Supplementary file 15 [file DataSheet1.DOCX]

The data file is to large to upload, please download from the Figshare link: <https://doi.org/10.6084/m9.figshare.16649887.v1> .
